# Design and Synthesis of New Acridone-Based Nitric Oxide Fluorescent Probe

**DOI:** 10.3390/molecules26144340

**Published:** 2021-07-17

**Authors:** Mikhail Panfilov, Darya Chernova, Irina Khalfina, Alexander Moskalensky, Aleksey Vorob’ev

**Affiliations:** 1 Novosibirsk Institute of Organic Chemistry, 9 Lavrentiev Avenue, 630090 Novosibirsk, Russia; panfilov@nioch.nsc.ru (M.P.); khalfina@nioch.nsc.ru (I.K.); 2 Department of Physics, Novosibirsk State University, 630090 Novosibirsk, Russia; che14darya@gmail.com (D.C.); sunmosk@mail.ru (A.M.)

**Keywords:** nitric oxide probes, 9(10*H*)acridone, fluorescent probes

## Abstract

Nitric oxide (NO) is an important signaling molecule involved in a wide range of physiological and pathological processes. Fluorescent imaging is a useful tool for monitoring NO concentration, which could be essential in various biological and biochemical studies. Here, we report the design of a novel small-molecule fluorescent probe based on 9(10*H*)acridone moiety for nitric oxide sensing. 7,8-Diamino-4-carboxy-10-methyl-9(10*H*)acridone reacts with NO in aqueous media in the presence of O_2_, yielding a corresponding triazole derivative with fivefold increased fluorescence intensity. The probe was shown to be capable of nitric oxide sensing in living Jurkat cells.

## 1. Introduction

Being a unique molecule, nitric oxide (II) plays a vital role in many physiological processes. Nowadays, nitric oxide has been shown to take part in neurotransmission, vasodilatation, smooth muscle tone regulation, immune system reaction, and so forth [1,2,3,4,5]. It means that lack or excess of it will inevitably lead to pathological states, such as cardiovascular disease, neurodegeneration, or even the formation of tumors [6]. Hence, monitoring the level and transformation pathways of nitric oxide is crucial for normal body functioning and for the further understanding of nitric oxide’s biological role.

**Figure 1:**
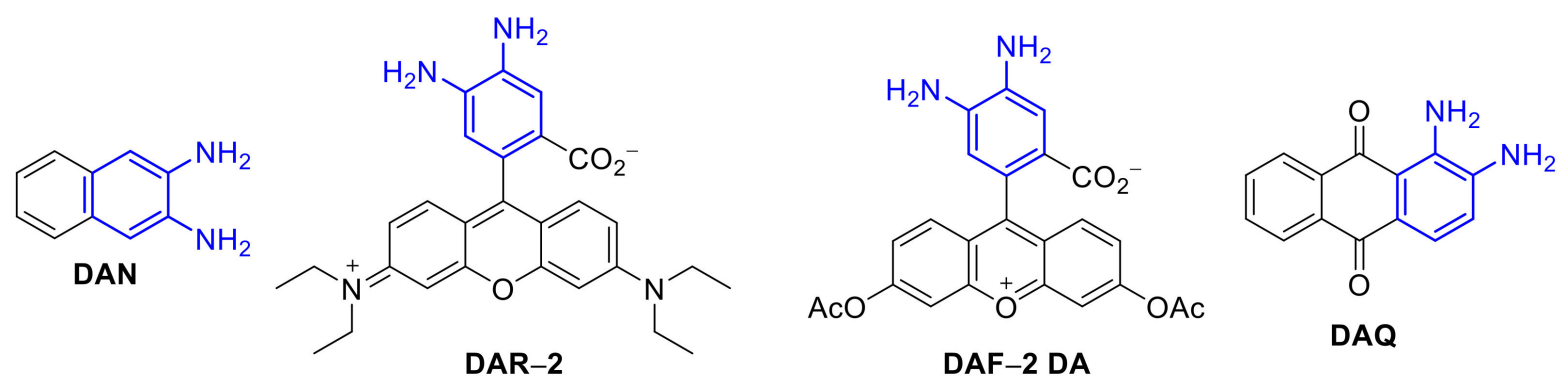
Examples of commercially available fluorescent nitric oxide probes.

The fluorescent microscopy technique, involving fluorescent probes specifically reacting with NO, allows one to achieve the abovementioned tasks. The design of the majority of such fluorescent probes is based on the conjugation of fluorophore with phenylene-1,2-diamine moiety, which undergoes oxygen-promoted reaction with NO forming the triazole ring. Such transformation causes significant growth of fluorescence quantum yield by blocking the photoinduced electron transfer [7,8]. To this day, a wide range of NO probes utilizing the abovementioned principle have been reported with BODIPY [9], coumarin [10,11], fluorescein [12], rhodamine [13,14], and other fluorophores. Several commercially available substances, such as DAN, DAR, or DAF (Figure 1), have found wide applications for the detection and imaging of NO in living cells [15]. 1,2-Diaminoanthraquinone (DAQ) is also a commercially available and cheap sensor that gives significant fluorescence intensity enhancement upon reaction with NO [16,17]. However, DAQ has several disadvantages, such as poor solubility in water and most organic solvents of a parent molecule and the triazole derivative and difficulties of modification. In order to expand the present range of fluorescent probes, we paid attention to acridone scaffold as the close analog of anthraquinone. Recently, several NO probes based on the acridine core has been reported [18,19]. Acridones are commonly available by intramolecular condensation of N-aryl-*ortho*-aminobenzoic acids [20] and could be easily modified at the N(10) atom [21,22]. We hypothesized that the incorporation of the *o*-diamine fragment along with the carboxylic group would improve water solubility.

## 2. Results and Discussion

### 2.1. Synthesis of 1,2-Diamino-10-(carboxymethyl)-9(10H)-acridone

**Scheme 1:**
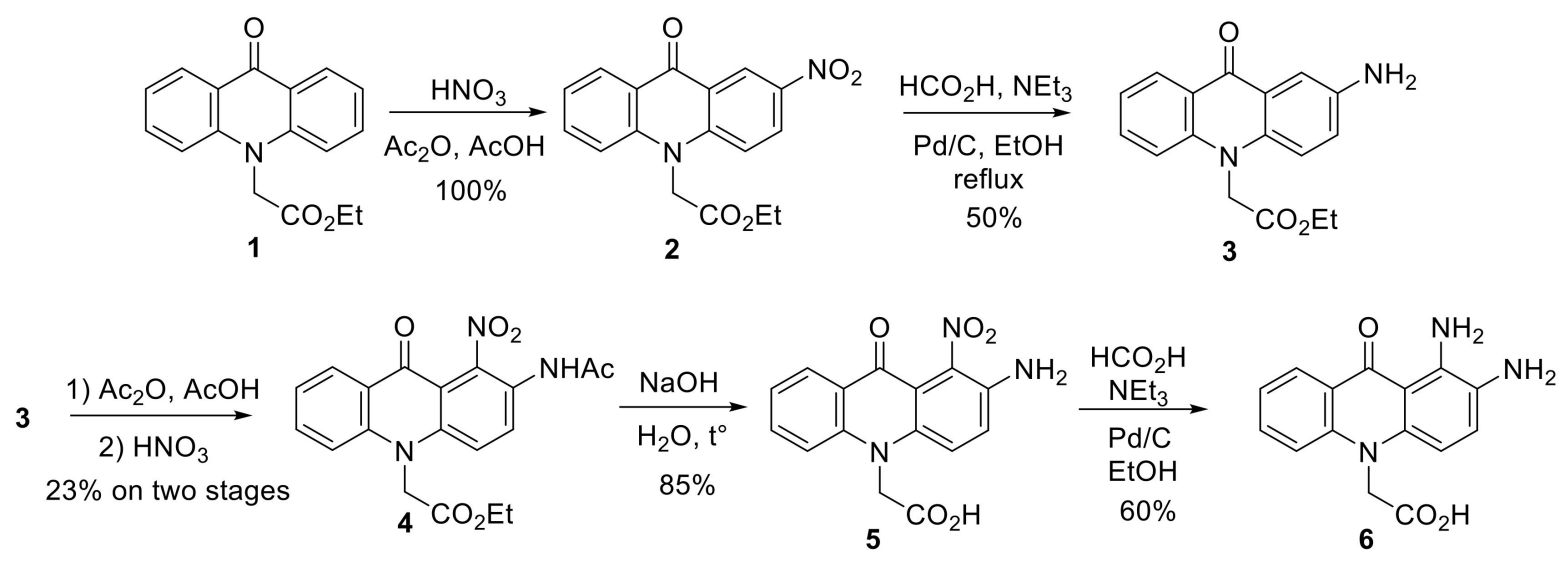
1,2-Diamino-10-(carboxymethyl)-9(10*H*)-acridone (**6**).

Scheme 1 illustrates our synthetic approach to 1,2-diamino-10-(carboxymethyl)-9(10*H*)-acridone. Nitration of 10-(carboxymethyl)-9(10*H*)acridone ethyl ester (**1**) with nitric acid in Ac_2_O/AcOH mixture quantitatively yielded nitro derivative **2**, which was reduced with formic acid over Pd/C in ethanol to give aminoacridone **3**. Application of hydrogen with Pd/C or Ni_Re_ as catalysts failed to give a satisfactory yield of **3**. Worse results were also obtained with N_2_H_4_ over Pd/C probably due to hydrazide formation. Acylation of **3** with Ac_2_O with subsequent nitration resulted in obtaining acridone **4**. Deprotection of the amino group by hot aqueous NaOH was accompanied by CO_2_Et group hydrolysis, leading to compound **5**, which was reduced to target diaminoacridone **6**.

### 2.2. Synthesis of 7,8-Diamino-4-carboxy-10-methyl-9(10H)acridone

**Scheme 2:**
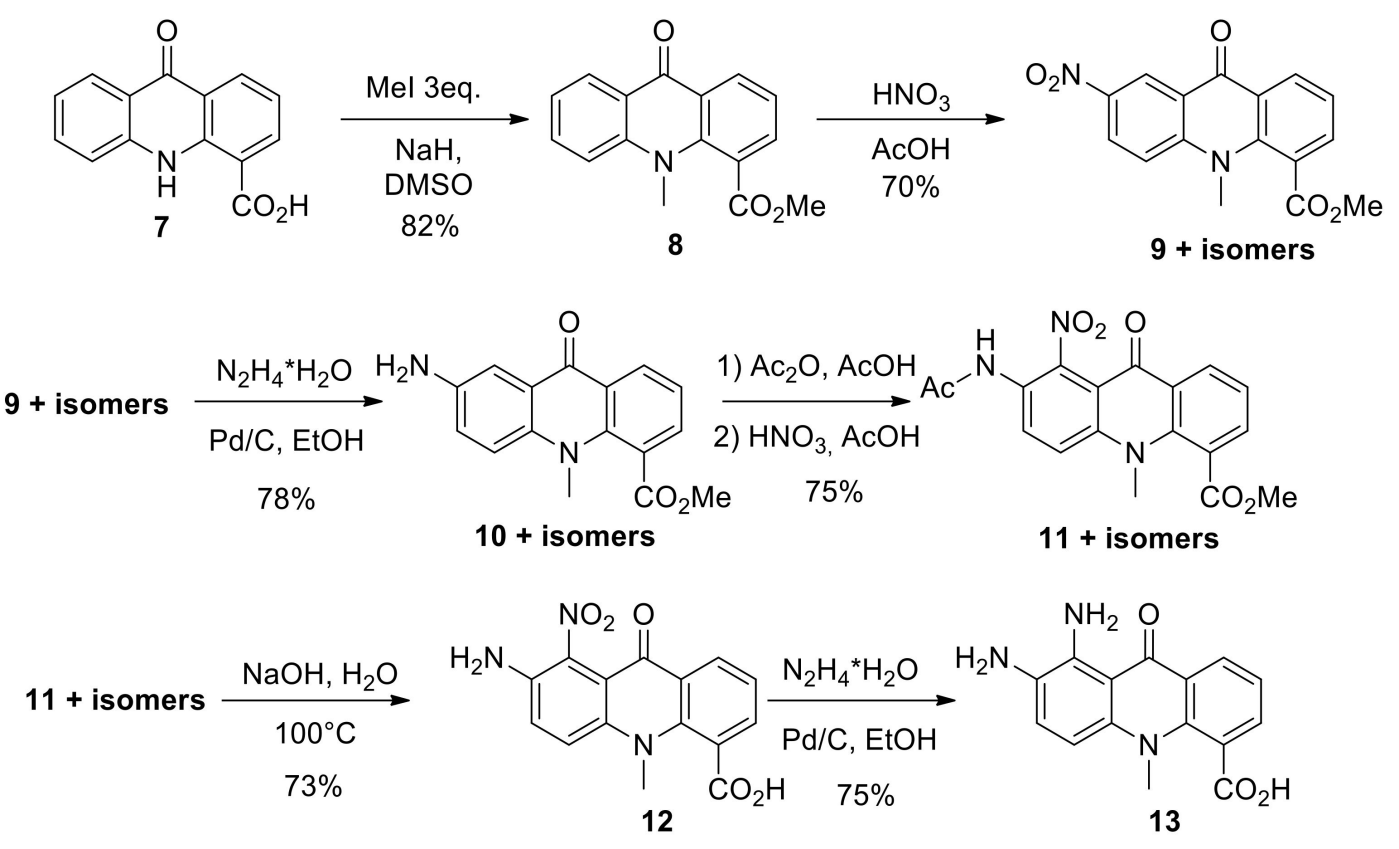
Synthesis of 7,8-diamino-4-carboxy-10-methyl-9(10*H*)acridone (**13**).

Synthesis of 7,8-diamino-10-methyl-9-oxo-9,10-dihydroacridine-4-carboxylic acid was carried out in a similar way starting from 9-oxo-9,10-dihydroacridine-4-carboxylic acid (**7**) (Scheme 2). Methylation of **7** with the MeI/NaH system in DMF resulted in obtaining *N*-methylated ester **8**. Ester **8** was nitrated with nitric acid in AcOH to give a mixture of three isomeric mononitro derivatives with nitroacridone **9** as the main isomer. This mixture was reduced without purification with hydrazine hydrate in the presence of Pd/C to obtain aminoacridone **10** with isomeric impurities. The amino group of compound **10** was then protected using Ac_2_O in acetic acid and nitrated in *one pot* mode with concentrated nitric acid to give **11**. Then, acridone **11** was hydrolyzed using a 4N solution of NaOH, resulting in compound **12**, which was purified from isomeric impurities by reprecipitation. Reduction of the nitro group in **12** by hydrazine hydrate over Pd/C led to 10-methyl-7,8-diamino-4-carboxy-9(10*H*)acridone (**13**).

### 2.3. Spectral Properties and NO Trapping

**Figure 2:**
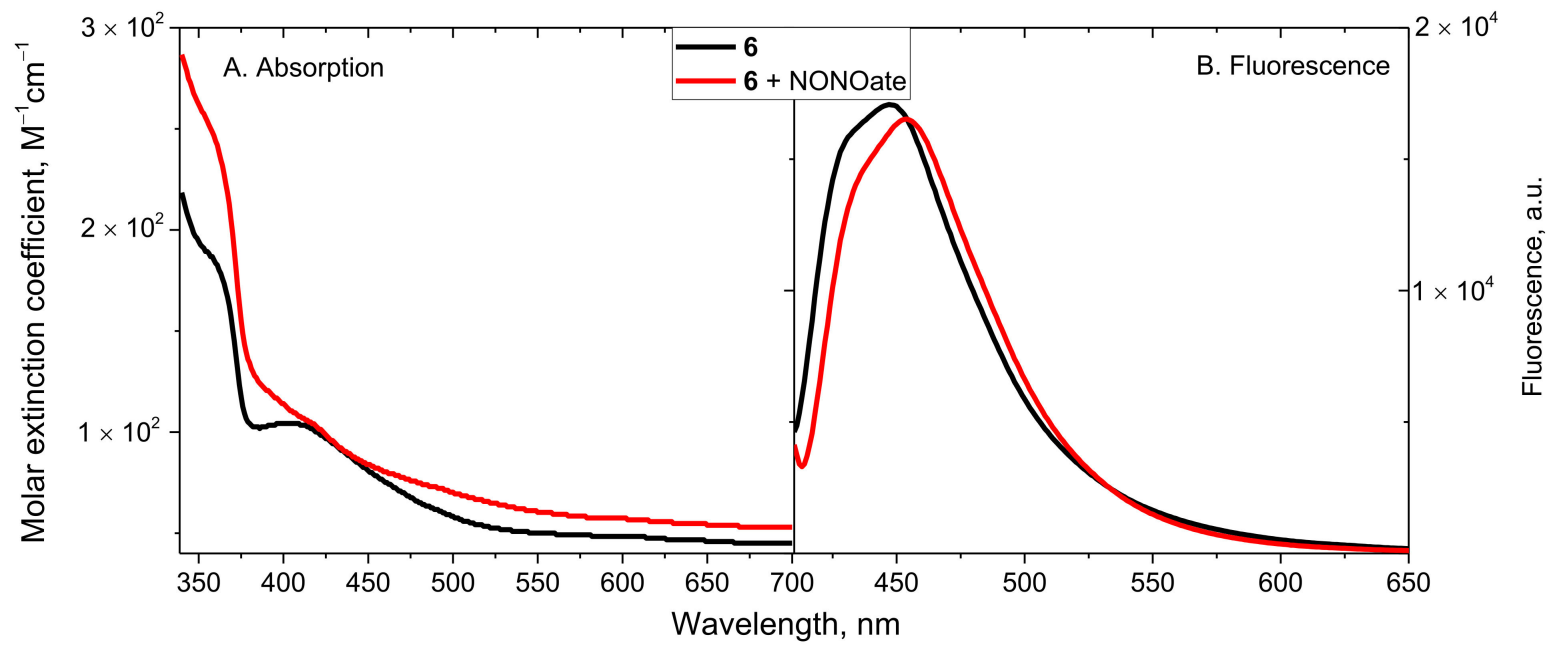
Absorption (**A**) and fluorescence (**B**) spectra of **6** before and after reaction with the excess of NONOate. Solvent: water + 1% DMSO. Fluorescence excitation: 405 nm.

Both diaminoacridones **6** and **13** were characterized by UV–VIS and fluorescence spectroscopy, and their ability of nitric oxide sensing was studied. Figure 2a shows the absorption spectra of **6** in aqueous solution with 1% DMSO before and after reaction with the excess of diethylamine NONOate diethylammonium salt (NONOate) for 10 min. Acridone **6** has low absorbance in the visible region, and spectral changes upon reaction with NONOate were insignificant. The changes in fluorescence maximum and intensity were also subtle (Figure 2b). Moreover, our attempts to obtain triazole by the diazotation of **6** were unsuccessful, and only complex reaction mixtures were obtained.

**Figure 3:**
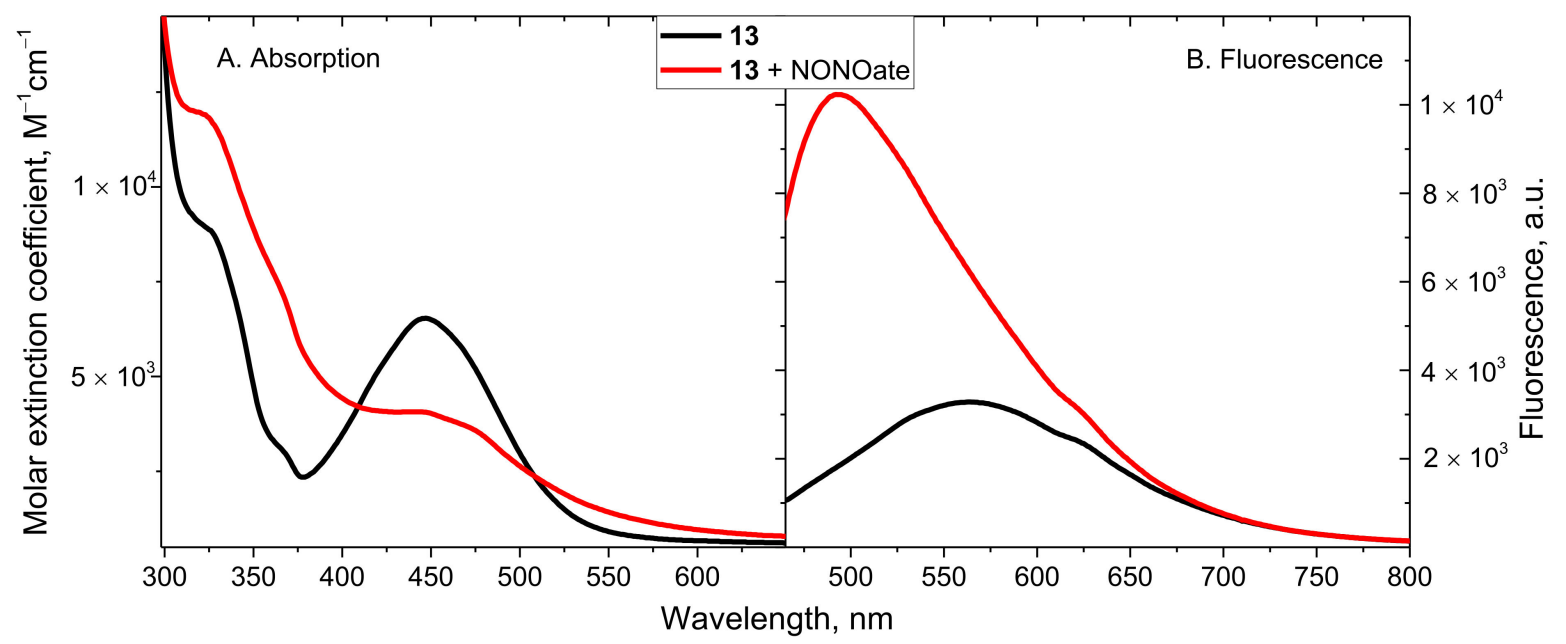
Absorption (**A**) and fluorescence (**B**) spectra of **13** before and after reaction with the excess of NONOate. Solvent: water + 1% DMSO. Fluorescence excitation: 450 nm.

**Scheme 3:**
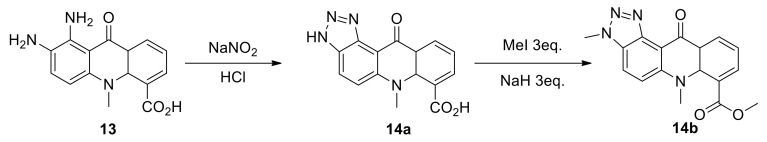
Proving the triazole ring formation of 10-methyl-7,8-diamino-4-carboxy-9(10*H*)acridone.

Compound **13** has an absorption maximum at 447 nm (Table 1), which falls within the most widespread excitation ranges of modern fluorescent microscopes (450–490 nm). The absorbance curve after the addition of NONOate significantly changed, indicating that structural changes occurred. As shown in Figure 3b, fluorescence intensity increased by a factor of 5 after reacting with nitric oxide, with the fluorescence maximum shifted to 495 nm from 564 nm. Triazole **14a**, which was obtained by the diazotation of **13** (Scheme 3), has weaker absorption in a visible region and a 7.6-fold increased fluorescence quantum yield compared with **13**. Unfortunately, molecular ion [**14a**]**^+∙^** was not observed by HRMS probably due to fast decomposition by the ejection of a N_2_ molecule, so we prepared the methylated analog **14b,** which has been well characterized by HRMS.

### 2.4. NO Imaging in Jurkat Cells

To demonstrate the applicability of **13** for the imaging of NO in living cells, we used the Jurkat cell line. A solution of 10 μM of **13** in water + 0.1% DMSO was added to the culture dish. After 15 min of incubation, cells were placed under a fluorescent microscope for observation. The standard filter set was used (excitation: 450–490 nm; emission: >515 nm).

**Figure 4:**
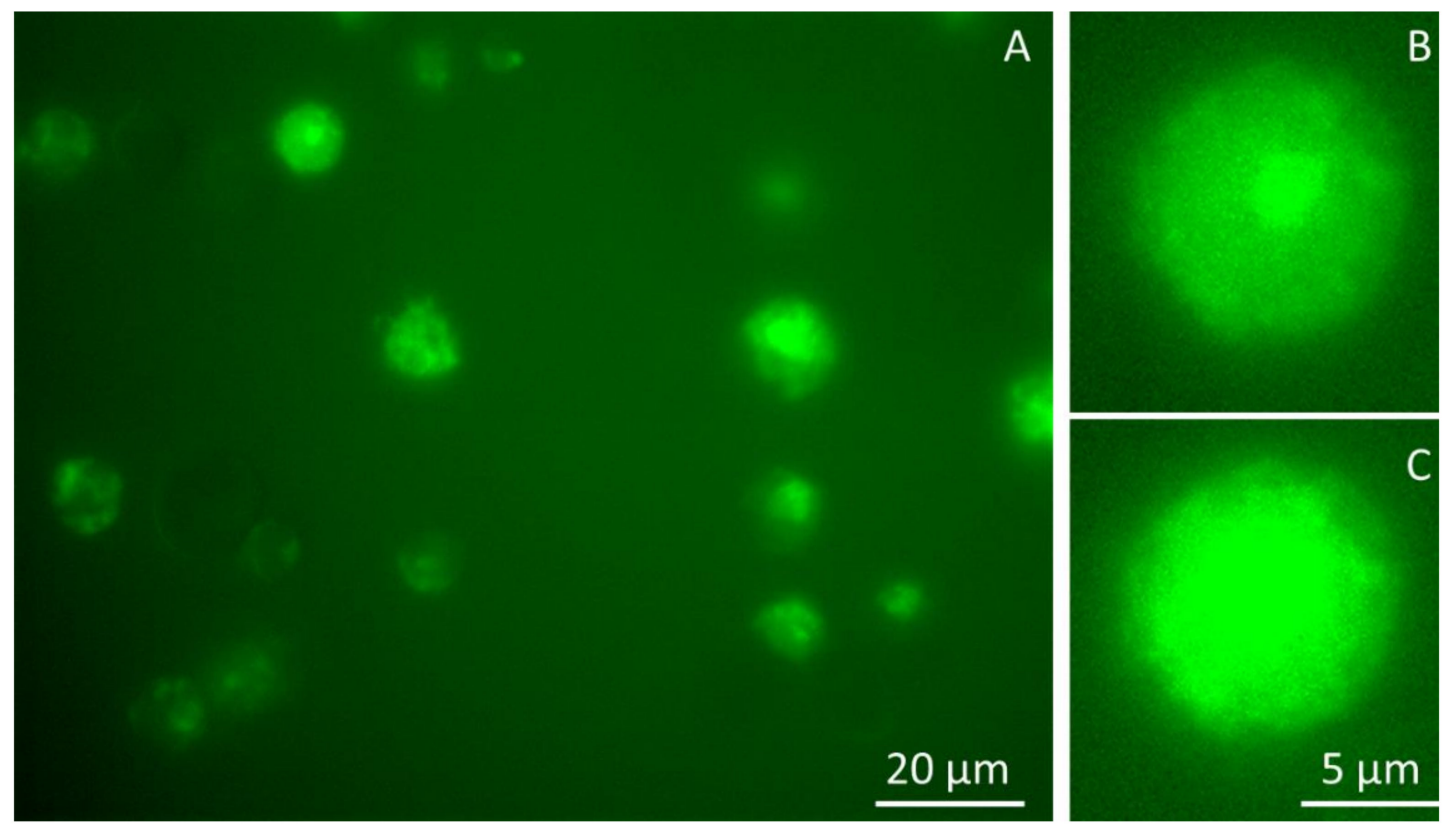
(**A**) Fluorescence of Jurkat cells incubated with **13** for 15 min. (**B**) The distribution of fluorescence is inhomogeneous. (**C**) Increased fluorescence 3 min after the addition of NONOate.

Figure 4A shows an image of Jurkat cells with bright fluorescence, which is the sign of **13** inside cells. The distribution of fluorescence in cells were inhomogeneous, as can be seen in Figure 4B. After the addition of 6 mM of NONOate, the fluorescence increase was visible. Figure 4C shows the same cell after 3 min. It justifies that **13** reacts with NO inside cells as well.

## 3. Materials and Methods

### 3.1. General Remarks

All chemicals were purchased from commercial sources and used without additional purification, unless otherwise noted. The progress of reactions was monitored by thin-layer chromatography (TLC) on Silica gel 60 F254 aluminum sheets. NMR spectra were recorded on Bruker Avance-300 (300.13 MHz for ^1^H) and Avance-400 (400.13 MHz for ^1^H, 100.62 MHz for ^13^C) spectrometers; chemical shifts of ^1^H and ^13^C{^1^H} are given in ppm, with solvent signals serving as the internal standard (^1^H = 7.24 ppm and ^13^C = 77.16 ppm for CDCl_3_; ^1^H = 2.50 ppm and ^13^C = 39.52 ppm for DMSO-*d*_6_). UV–VIS spectra were registered on a Shimadzu UV-1900 spectrophotometer, and fluorescence spectra were obtained on a Shimadzu RF-6000 fluorometer for 10^−4^ M solutions in 1% DMSO in H_2_O. Quantum yields were determined according the standard procedure with rhodamine 6G (10^−6^ M solution in H_2_O) as the standard. Masses of molecular ions were determined by high-resolution mass spectrometry (HRMS) by means of a DFS Thermo Scientific instrument (EI, 70 eV) or by using a Bruker micrOTOF-Q for ESI experiments. An inverted fluorescent microscope Carl Zeiss Axio Vert.A1 equipped with an Axiocam 503 mono high-sensitive camera was used in this study for the observation of Jurkat cells. The cells were grown in standard conditions: IMDM basic medium supplemented with L-glutamine, 25 mM HEPES, fetal bovine serum, and gentamicin in an incubator at 37 °C and 5% CO_2_. 9-Acridone and 4-carboxy-9(10*H*)acridone were synthesized according to the literature methods [23,24]. The spectra for synthesized compounds can be found in the Supplementary Materials.

### 3.2. Synthesis

#### 3.2.1. Synthesis of 1,2-Diamino-10-(carboxymethyl)-9(10*H*)-acridone (**6**)

*Synthesis of 10-(carboxymethyl)-9(10H)acridone ethyl ester* (**1**). To a stirred solution of 1.87 g of 9-acridone (9.57 mmol) in 10 mL of DMF at 60 ^°^C, 0.57 g (14.4 mmol) of NaH was added. The mixture was stirred for additional 15 min, and then 2.07 g (12.5 mmol) of ethyl bromoacetate was added. The reaction mixture was stirred for 3 h, cooled to room temperature, and poured in water. The obtained precipitate was filtered off and washed a few times with water. The obtained solid was recrystallized using ethanol as a solvent. Pale yellow solid. Yield, 1.48 g (55%). ^1^H NMR (300 MHz, DMSO-*d*_6_) δ 1.20 (t, *J* = 7.1 Hz, 3H), 4.18 (q, *J* = 7.1 Hz, 2H), 5.40 (s, 2H), 7.33 (t, *J* = 7.4 Hz, 2H), 7.62 (d, *J* = 8.7 Hz, 2H), 7.78 (t, *J* = 8.7 Hz, 2H), 8.32 (dd, *J* = 8.0, 1.7 Hz, 2H). An NMR spectrum is in good agreement with a literature one [25].

*Synthesis of 2-nitro-10-(carboxymethyl)-9(10H)acridone ethyl ester* (**2**). An amount of 0.670 mL (15 mmol) of nitric acid was added to a mixture of 1.4 g (5 mmol) of compound **1,** followed by 3.3 mL (35 mmol) of acetic anhydride in 15 mL of acetic acid. The mixture was stirred for 1.5 h at 60 ^°^C. The resulting mixture was cooled to room temperature and neutralized with 10% NaOH solution. The formed precipitate was filtered off and washed a few times with water. Yellow solid. Yield, 1.63 g (100%). M.p. 196.5–201.4 ^°^C. ^1^H NMR (300 MHz, DMSO-*d*_6_) δ 1.22 (t, *J* = 7.1 Hz, 3H), 4.20 (q, *J* = 7.1 Hz, 2H), 5.50 (s, 2H), 7.44 (t, *J* = 7.5 Hz, 1H), 7.70 (d, *J* = 8.7 Hz, 1H), 7.82—7.91 (m, 2H), 8.33 (dd, *J* = 8.0, 1.7 Hz, 1H), 8.48 (dd, *J* = 9.5, 2.9 Hz, 1H), 9.01 (d, *J* = 2.9 Hz, 1H); ^13^C NMR (101 MHz, DMSO-*d*_6_) δ 14.1, 47.6, 61.4, 115.8, 117.4, 120.5, 121.1, 121.6, 121.8, 126.0, 126.7, 133.5, 134.4, 140.9, 142.1, 168.7, 176.8. HRMS (EI): calc for C_17_H_14_N_2_O_5_ 326.0897 *m*/*z*; found 326.0901 *m*/*z*.

*Synthesis of 2-amino-10-(carboxymethyl)-9(10H)acridone ethyl ester* (**3**). A mixture of 0.75 g (23 mmol) of compound **2**, 0.080 g of Pd/C, 1.3 mL (0.035 mol) of formic acid, and 4.2 mL (34.5 mol) of TEA in 20 mL of ethanol was refluxed for 2 h. The resulting mixture was filtered off while hot, and Pd/C was washed with hot ethanol. Ethanol was evaporated under low pressure. The residue was recrystallized using ethanol. Orange solid. Yield, 0.34 g (50%). ^1^H NMR (400 MHz, DMSO-d_6_) δ 1.23 (t, *J* = 7.1 Hz, 4H), 4.20 (q, *J* = 7.1 Hz, 2H), 5.34 (s, 2H), 7.17 (dd, *J* = 9.1, 2.8 Hz, 1H), 7.25 (m, 1H), 7.43 (d, *J* = 9.1 Hz, 1H), 7.51 (d, *J* = 2.8 Hz, 1H), 7.56 (d, *J* = 8.8 Hz, 1H), 7.71 (m, 1H), 8.30 (dd, *J* = 8.0, 1.7 Hz, 1H); ^13^C NMR (101 MHz,) δ 14.1, 47.4, 61.2, 107.2, 115.2, 116.5, 120.5, 120.5, 122.9, 123.2, 126.6, 133.3, 134.0, 141.4, 143.8, 168.8, 176.2. HRMS (ESI): calc. for C_17_H_17_O_3_N_2_^+^, 297.1239 *m/z*; found 297.1235 *m*/*z*.

*Synthesis of 2-acetamide-1-nitro-10-(carboxymethyl)-9(10H)acridone ethyl ester* (**4**). An amount of 0.34 g of compound **3** (1.27 mmol) was added to a mixture of 10 mL of acetic acid and 0.6 mL of acetic anhydride. The reaction mixture was stirred for 1.5 h at 60 ^°^C. After the reaction completion, the mixture was cooled to room temperature and diluted with water until the precipitate was formed. The formed precipitate was filtered off and washed with water. Yellow solid, used without purification. Yield, 0.237 g (55%). ^1^H NMR (300 MHz, DMSO-*d*_6_) δ 1.24 (t, *J* = 7.1 Hz, 3H), 2.08 (s, 3H), 4.21 (q, *J* = 7.1 Hz, 2H), 5.42 (d, *J* = 3.2 Hz, 2H), 7.29–7.38 (m, 1H), 7.58–7.68 (m, 2H), 7.75–7.84 (m, 1H), 8.01 (dd, *J* = 9.3, 2.7 Hz, 1H), 8.34 (dd, *J* = 8.0, 1.7 Hz, 1H), 8.55 (d, *J* = 2.6 Hz, 1H), 10.20 (s, 1H); ^13^C NMR (75 MHz, DMSO-*d*_6_) δ 14.4, 24.3, 47.83, 61.7, 115.5, 115.91, 116.7, 121.4, 121.8, 122.1, 126.7, 127.0, 134.1, 134.4, 138.5, 142.1, 168.7, 169.0, 176.8.

A mixture of the obtained 0.2 g (0.59 mmol) of acetamide, 0.3 mL (3 mmol) of acetic anhydride, and 0.06 mL (1.18 mmol) of nitric acid in 5 mL of acetic acid was stirred at 60 ^°^C until reaction completion by TLC. The resulting mixture was cooled to room temperature, poured into water, and neutralized with sodium hydroxide solution. The formed precipitate was filtered off and washed a few times with water. The obtained solid was recrystallized using ethanol. Yellow solid. Yield, 0.097 g (40%). ^1^H NMR (300 MHz, CDCl_3_) δ 0.99 (t, *J* = 7.1 Hz, 3H), 1.84 (s, 3H), 3.98 (q, *J* = 7.2 Hz, 2H), 4.87 (s, 2H), 6.95–7.11 (m, 2H), 7.19–7.27 (m, 1H), 7.44 (s, 1H), 7.75 (d, *J* = 9.4 Hz, 1H), 8.02 (d, *J* = 7.7 Hz, 1H), 9.09 (s, 1H); ^13^C NMR (101 MHz, CDCl_3_) δ 13.3, 22.4, 47.9, 61.4112.0, 114.0, 116.2, 121.4, 122.0, 123.5, 126.5, 132.5, 134.0, 139.2, 140.6, 142.0, 166.8, 169.6, 173.6. HRMS (EI): calc for C_19_H_17_N_3_O_6_ 383.1117 *m*/*z*; found 383.1119 *m*/*z*.

*Synthesis of 2-amino-1-nitro-10-(carboxymethyl)-9(10H)-acridone* (**5**). A solution of 1 g (25.3 mmol) of sodium hydroxide in 7 mL of water was added to 0.097 g (0.253 mmol) of compound **4**. The reaction mixture was refluxed, until the starting sediment was dissolved. The resulting solution was cooled to room temperature and acidified with concentrated hydrochloric acid until the precipitate was formed. The precipitate was filtered off and washed a few times with water. Dark brown solid. Yield, 0.75 g (95%). ^1^H NMR (300 MHz, DMSO-*d*_6_) δ = 5.29 (s, 2H), 7.36 (t, *J* = 7.6, 1H), 7.58 (d, *J* = 9.5, 1H), 7.68 (d, *J* = 8.8, 1H), 7.81 (m, 2H), 8.23 (d, *J* = 8.0, 1H); ^13^C NMR (101 MHz, DMSO-*d*_6_) δ 48.0, 113.0, 115.8, 119.1, 120.9, 122.0, 124.9, 126.3, 134.6, 135.1, 135.4, 141.4, 144.0, 169.7, 173.6. HRMS (ESI): calc. for C_15_H_10_N_3_O_5_^−^, 312.0620 *m*/*z*; found 312.0626 *m*/*z*.

*Synthesis of 1,2-diamino-10-(carboxymethyl)-9(10H)-acridone* (**6**). A mixture of 0.040 g (0.13 mmol) of compound **5**, 0.072 mL (1.92 mmol) of formic acid, 0.230 mL of TEA, and 0.02 g of Pd/C in 10 mL of ethanol was refluxed for 2 h. The resulting mixture was filtered off while hot, and Pd/C was washed with hot ethanol. The solvent was evaporated at low pressure. The residue was diluted with 5% hydrochloric solution and neutralized with 5% NaHCO_3_ solution. The formed precipitate was filtered off and washed with water. Dark solid. Yield, 0.021 g (60%). ^1^H NMR (400 MHz, DMSO-*d*_6_) δ 5.05 (s, 2H), 6.34 (d, *J* = 8.8 Hz, 1H), 7.07 (d, *J* = 8.7 Hz, 1H), 7.20 (t, *J* = 7.5 Hz, 1H), 7.47 (d, *J* = 8.7 Hz, 1H), 7.67 (t, *J* = 7.9 Hz, 1H), 8.25 (d, *J* = 8.0 Hz, 1H); ^13^C NMR (75 MHz, DMSO-*d*_6_) δ 47.8, 97.9, 107.9, 114.8, 119.7, 120.1, 121.0, 126.3, 133.5, 136.7, 137.0, 140.1, 141.8, 170.2, 179.9. HRMS (ESI): calc. for C_15_H_14_N_3_O_3_^−^, 282.0879 *m*/*z*; found 282.0877 *m*/*z*.

#### 3.2.2. Synthesis 7,8-Diamino-10-methyl-9-oxo-9,10-dihydroacridine-4-carboxylic Acid (**13**)

*Synthesis of methyl 10-methyl-9-oxo-9,10-dihydroacridine-4-carboxylate* (**8**). An amount of 2.3 g (9.6 mmol) of 4-carboxy-(9,10H)acridone was suspended in 30 mL of DMF. Then 1.52 g (28.8 mmol) of NaH was slowly added with continuous stirring. After the addition of NaH, 4.1 g (28.8 mmol) of MeI was added dropwise to the mixture. The reaction mixture was stirred for 2 h at room temperature until completion by TLC. The reaction mixture was diluted with water until the residue was formed. The obtained residue was filtrated and washed a few times with water. If the purity of the product was not satisfying, it could be recrystallized from ethanol. Green solid. Yield, 2.1 g (82%). M.p. 164.8 ^°^C. ^1^H NMR (400 MHz, CDCl_3_) δ = 3.69 (s, 3H), 3.98 (s, 3H), 7.29 (m, 2H), 7.44 (d, *J* = 8.5, 1H), 7.71 (ddd, *J* = 8.7, 7.0, 1.7, 1H), 8.02 (dd, *J* = 7.4, 1.8, 1H), 8.44 (dd, *J* = 8.0, 1.7, 1H), 8.63 (dd, *J* = 7.9, 1.8, 1H); ^13^C NMR (126 MHz, CDCl_3_) δ 41.6, 52.6, 116.4, 120.4, 121.4, 122.1, 122.9, 124.6, 127.1, 131.1, 134.0, 136.0, 143.0, 144.2, 168.3, 177.7. HRMS (EI): calc for C_16_H_13_NO_3_ 267.0890 *m*/*z*; found 267.0892 *m*/*z*.

*Synthesis of methyl 10-methyl-7-nitro-9-oxo-9,10-dihydroacridine-4-carboxylate* (**9**). An amount of 2 g (7.5 mmol) of compound **8** was suspended in 45 mL of glacial acetic acid. The mixture was heated to 60 ^°^C, and a mixture of 6.67 mL of nitric acid and 6.67 mL of acetic acid was added. The reaction was stirred until completion by TLC. The reaction mixture was poured in water with ice. The obtained precipitate was filtrated and thoroughly washed with water. The obtained solid contained 2-nitro isomer and traces of 5-nitro isomer. Yellow solid, used without purification. Yield of all isomers, 1.68 g (72%). ^1^H NMR (400 MHz, DMSO-*d*_6_) δ = 3.72 (d, *J* = 3.4, 3H), 3.98 (s, 3H), 7.49 (m, 1H), 7.91 (d, *J* = 9.3, 1H), 8.12 (dd, *J* = 7.4, 1.8, 1H), 8.44 (dd, *J* = 8.0, 1.8, 1H), 8.55 (dd, *J* = 9.4, 2.8, 1H), 8.93 (d, *J* = 2.8, 1H).

*Synthesis of methyl 7-amino-10-methyl-9-oxo-9,10-dihydroacridine-4-carboxylate* (**10**). A mixture of 1.25 g (0.004 mol) of **9**, 0.43 g of Pd/C, and 2 mL (0.04 mol) of N_2_H_4_·H_2_O in 75 mL EtOH was refluxed with stirring for 1 h. Then the reaction mixture was filtrated while hot, and Pd/C was washed a few times with hot ethanol. The filtrate was evaporated in vacuo, and the obtained precipitate was dried on air. Orange solid, which contained impurities (approximately 20%), used without purification. Yield, 0.88 g (78%). ^1^H NMR (300 MHz, DMSO-*d*_6_) δ = 3.60 (s, 3H), 3.93 (s, 3H), 7.21 (m, 1H), 7.28 (d, *J* = 7.5, 1H), 7.42 (d, *J* = 2.8, 1H), 7.50 (d, *J* = 9.1, 1H), 7.99 (dd, *J* = 7.3, 1.8, 1H), 8.43 (dd, *J* = 7.9, 1.8, 1H).

*Synthesis of methyl 7-acetamido-10-methyl-8-nitro-9-oxo-9,10-dihydroacridine-4-carboxylate* (**11**). To a solution of 0.6 g (2.13 mmol) of **10** in 12 mL of glacial acetic acid, 1 mL of Ac_2_O was added. The reaction mixture was stirred at 60 ^°^C for 1 h, and then a mixture of 1.9 mL of nitric acid and 1.9 mL of glacial acetic acid was added. The reaction was monitored by TLC, until initial the **10** disappeared. After completion, the reaction mixture was poured in water with ice, and the formed precipitate was filtrated and thoroughly washed with water. Yellow solid with impurities (approximately 20%), used without purification. Yield, 0.58 g (74%). ^1^H NMR (400 MHz, DMSO-*d*_6_) δ = 2.05 (s, 3H), 3.71 (s, 3H), 3.97 (s, 3H), 7.46 (t, *J* = 7.7, 1H), 8.00 (m, 2H), 8.12 (d, *J* = 6.8, 1H), 8.35 (d, *J* = 7.8, 1H), 9.97 (s, 1H).

*Synthesis of 7-amino-10-methyl-8-nitro-9-oxo-9,10-dihydroacridine-4-carboxylic acid* (**12**). An amount of 0.560 g of **11** was added to 37.5 mL of 4N NaOH solution and refluxed until complete dissolution of the initial precipitate. The mixture was cooled to room temperature and acidified with HCl to pH ~ 7. The formed precipitate was filtrated and thoroughly washed with water. Dark brown solid, obtained as pure. Yield, 0.347 g (73%). Decomposes without melting at >150 ^°^C. ^1^H NMR (400 MHz, DMSO-*d*_6_) δ = 3.80 (s, 3H), 7.43 (t, *J* = 7.6, 1H), 7.67 (d, *J* = 9.3, 1H), 7.94 (d, *J* = 9.4, 1H), 8.15 (d, *J* = 7.1, 1H), 8.37 (d, *J* = 7.8, 1H), 11.18 (s, 1H), 13.54 (s, 2H); ^13^C NMR (75 MHz, DMSO-*d*_6_) δ 42.6, 113.7, 120.9, 121.3, 123.3, 123.7, 125.0, 129.2, 134.6, 135.8, 137.2, 141.5, 144.3, 168.9, 173.5. HRMS (ESI): calc. for C_15_H_10_N_3_O_5_^−^, 312.0620 *m*/*z*; found 312.0624 *m*/*z*.

*Synthesis of 7,8-diamino-10-methyl-9-oxo-9,10-dihydroacridine-4-carboxylic acid* (**13**). A mixture of 0.432 g (1.38 mmol) of **12**, 0.15 g of Pd/C, and 0.7 mL (0.0138 mol) of N_2_H_4_·H_2_O in 50 mL of EtOH was refluxed with stirring for 1 h. Then the reaction mixture was filtrated while hot, and Pd/C was washed a few times with hot ethanol. The filtrate was evaporated in vacuo, and the obtained precipitate was diluted with 5 mL of water and acidified with HCl to pH ~ 7. The formed precipitate was filtered off and washed with water. Brown solid. Yield, 0.291 g (74%). ^1^H NMR (300 MHz, DMSO-*d*_6_) δ = 3.54 (s, 3H), 6.47 (d, *J* = 8.7, 1H), 7.11 (d, *J* = 8.6, 1H), 7.21 (t, *J* = 7.6, 1H), 7.96 (dd, *J* = 7.3, 1.8, 1H), 8.35 (dd, *J* = 7.9, 1.8, 1H), 9.37 (s, 1H); ^13^C NMR (75 MHz, DMSO-*d*_6_) δ 42.2, 100.0, 108.1, 119.3, 119.7, 122.2, 123.3, 129.2, 135.0, 137.2, 138.6, 140.0, 142.0, 169.2, 179.5. HRMS (ESI): calc. for C_15_H_14_N_3_O_3_^−^, 282.0879 *m*/*z*; found 282.0883 *m*/*z*.

#### 3.2.3. Diazotation of Diaminoacridone **13**

*Synthesis of 6-methyl-11-oxo-6,11-dihydro-3H-[1,2,3]triazolo[4,5-a]acridine-7-carboxylic acid* (**14a**). To 3 mL of the cooled concentrated hydrochloric acid, 0.015 g (0.21 mmol) of NaNO_2_ was added. After a pale-yellow solution occurred, 0.06 g (0.21 mmol) of **6** was added. The solution was stirred until an orange precipitate was formed. The reaction mixture was diluted with water, and the solid was filtered off and washed with water. Orange solid, obtained pure. Yield, 0.048 g (77%). ^1^H NMR (400 MHz, DMSO-*d*_6_) δ = 3.79 (s, 3H), 7.07 (d, *J* = 10.1, 1H), 7.43 (t, *J* = 7.6, 1H), 8.07 (m, 2H), 8.32 (dd, *J* = 8.0, 1.7, 1H); ^13^C NMR (75 MHz, DMSO-*d*_6_) δ 42.1, 74.9, 112.9, 122.5, 122.9, 124.5, 128.5, 130.5, 131.3, 135.1, 135.6, 140.4, 168.8, 171.1, 177.0.

*Synthesis of methyl 3,6-dimethyl-11-oxo-6,11-dihydro-3H-[1,2,3]triazolo[4,5-a]acridine-7-carboxylate* (**14b**). An amount of 0.090 g (0.31 mmol) of **14a** was suspended in 5 mL of DMF. With cooling and stirring, 0.035 g (0.92 mmol) of NaH was added to the reaction mixture. The mixture was stirred for 15 min, and then 0.130 g (0.92 mmol) of MeI was added dropwise. The reaction was stirred for another 3 h and diluted with water, and the formed precipitate was filtered off and washed with water. Dark solid, obtained pure. Yield, 0.010 g (10%). ^1^H NMR (400 MHz, DMSO-*d*_6_) δ = 3.30 (s, 3H), 3.74 (s, 3H), 3.96 (s, 3H), 7.10 (d, *J* = 10.1, 1H), 7.47 (t, *J* = 7.6, 1H), 8.08 (m, 2H), 8.41 (dd, *J* = 8.1, 1.8, 1H). HRMS (EI): calc. for C_17_H_14_O_3_N_4_, 322.1060 *m*/*z*; found 322.1066 *m*/*z*.

## 4. Conclusions

In summary, a new fluorescent probe, 7,8-diamino-4-carboxy-10-methyl-9(10*H*)acridone, for nitric oxide sensing based on acridone moiety has been reported. Its spectroscopic properties fall within the most widespread excitation ranges of modern fluorescent microscopes. The probe has shown the ability to trap nitric oxide with a fluorescence increase. Furthermore, the probe has been shown to be capable of sensing nitric oxide in Jurkat living cells. In contrast to a commercial **DAQ** probe, the described diaminoacridone demonstrates solubility in water media. Furthermore, the suggested synthetic protocol allows simple modification at the N(10) atom.

## Figures and Tables

**Table 1:** Spectral properties of **13** and **14a**.

	Absorption Maximum λ_max_	Molar Extinction Coefficient ε(λ_max_)	Emission Maximum	Fluorescence Quantum Yield
**13**	447 nm	6500 M^−1^cm^−1^	564 nm	0.19%
**14a**	440 nm	4100 M^−1^cm^−1^	494 nm	1.45%

## Data Availability

Not applicable.

## Supplementary Materials

^1^H and ^13^C NMR spectra of the obtained compounds.
