# Supplementary material for: Design and Synthesis of New Acridone-Based Nitric Oxide Fluorescent Probe
Source: Molecules. 2021 Jul 17;26(14):4340. doi: 10.3390/molecules26144340 (PMC8307419; doi:10.3390/molecules26144340)
Supplement: Supplementary file 1 [file molecules-26-04340-s001.zip › molecules-1281988 supplimentary (3).pdf]

Supplementary materials

Design and synthesis of new acridone-based nitric oxide fluorescent probe

Mikhail Panfilov [1], Daria Chernova [2], Irina Khalfina [1,2], Alexander Moskalensky [2] and Alexey Vorob'ev [1,2]*

[1] Novosibirsk Institute of Organic Chemistry, Russian Federation, 630090, Novosibirsk, 9 Lavrentiev avenue; e-mail@e-mail.com
[2] Affiliation 2; e-mail@e-mail.com
* Correspondence: vor@nioch.nsc.ru; Tel.: +7 952 9488714

^{1}H spectrum of compound **1** ____ 2
^{1}H spectrum of compound **2** ____ 3
^{13}C spectrum of compound **2** ____ 4
^{1}H spectrum of compound **3** ____ 5
^{13}C spectrum of compound **3** ____ 6
^{1}H spectrum of compound **4** ____ 7
^{13}C spectrum of compound **4** ____ 8
^{1}H spectrum of compound **5** ____ 9
^{13}C spectrum of compound **5** ____ 10
^{1}H spectrum of compound **6** ____ 11
^{13}C spectrum of compound **6** ____ 12
^{1}H spectrum of compound **8** ____ 13
^{13}C spectrum of compound **8** ____ 14
^{1}H spectrum of compound **9** ____ 15
^{1}H spectrum of compound **10** ____ 16
^{1}H spectrum of compound **11** ____ 17
^{1}H spectrum of compound **12** ____ 18
^{13}C spectrum of compound **12** ____ 19
^{1}H spectrum of compound **13** ____ 20
^{13}C spectrum of compound **13** ____ 21
^{1}H spectrum of compound **14a** ____ 22
^{13}C spectrum of compound **14a** ____ 23
^{1}H spectrum of compound **14b** ____ 24

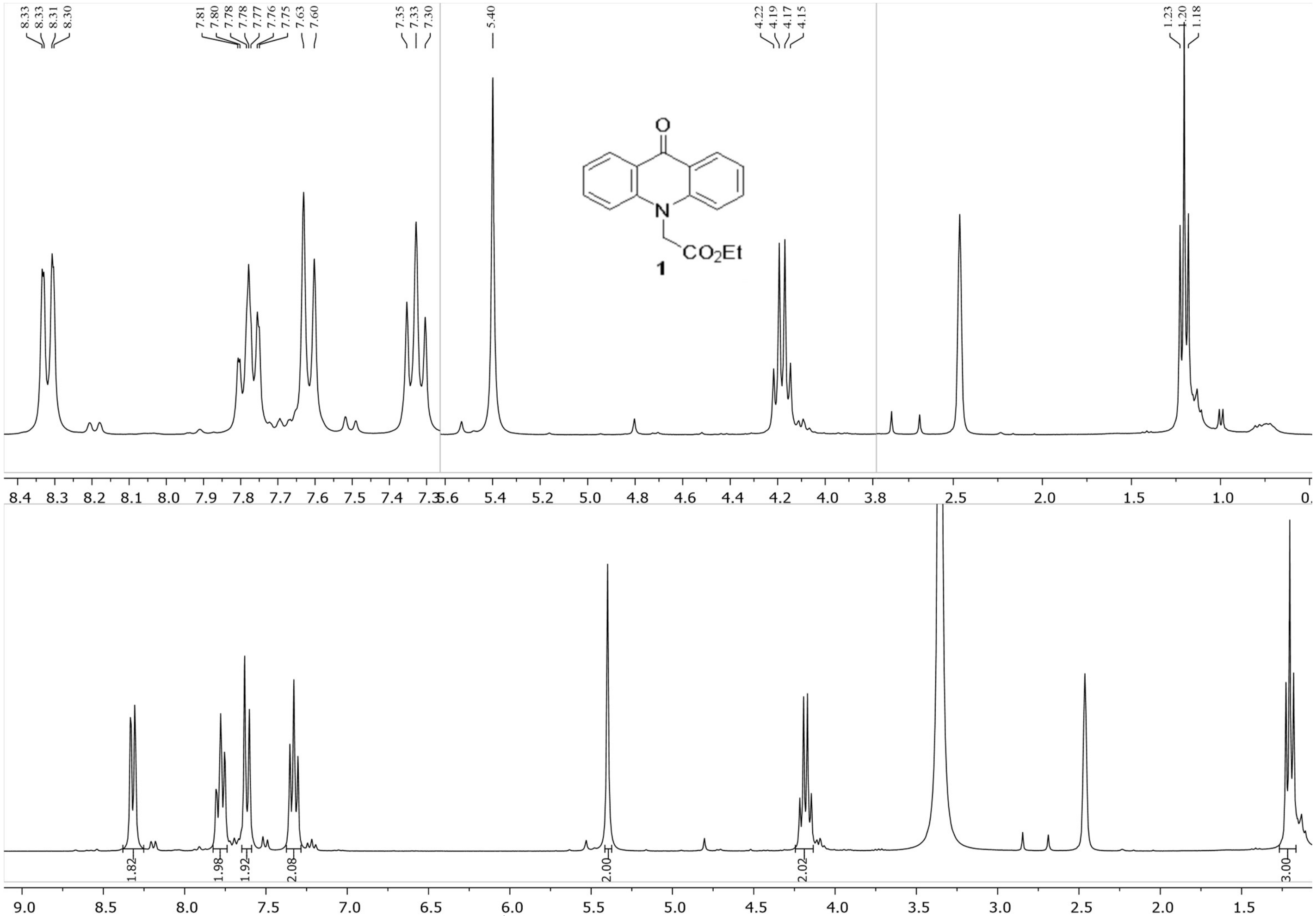

^{1}H spectrum of compound **1**

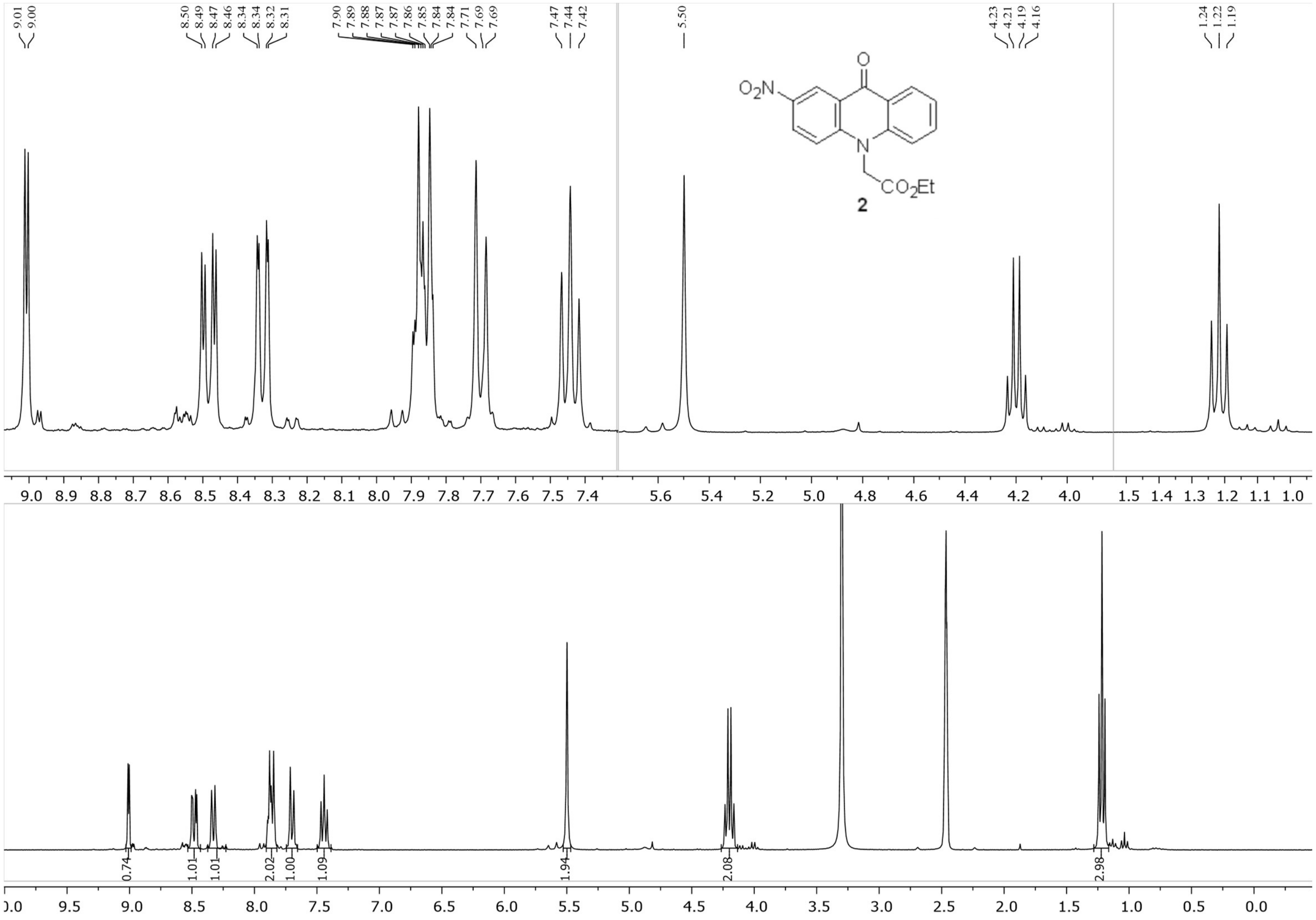

^{1}H spectrum of compound **2**

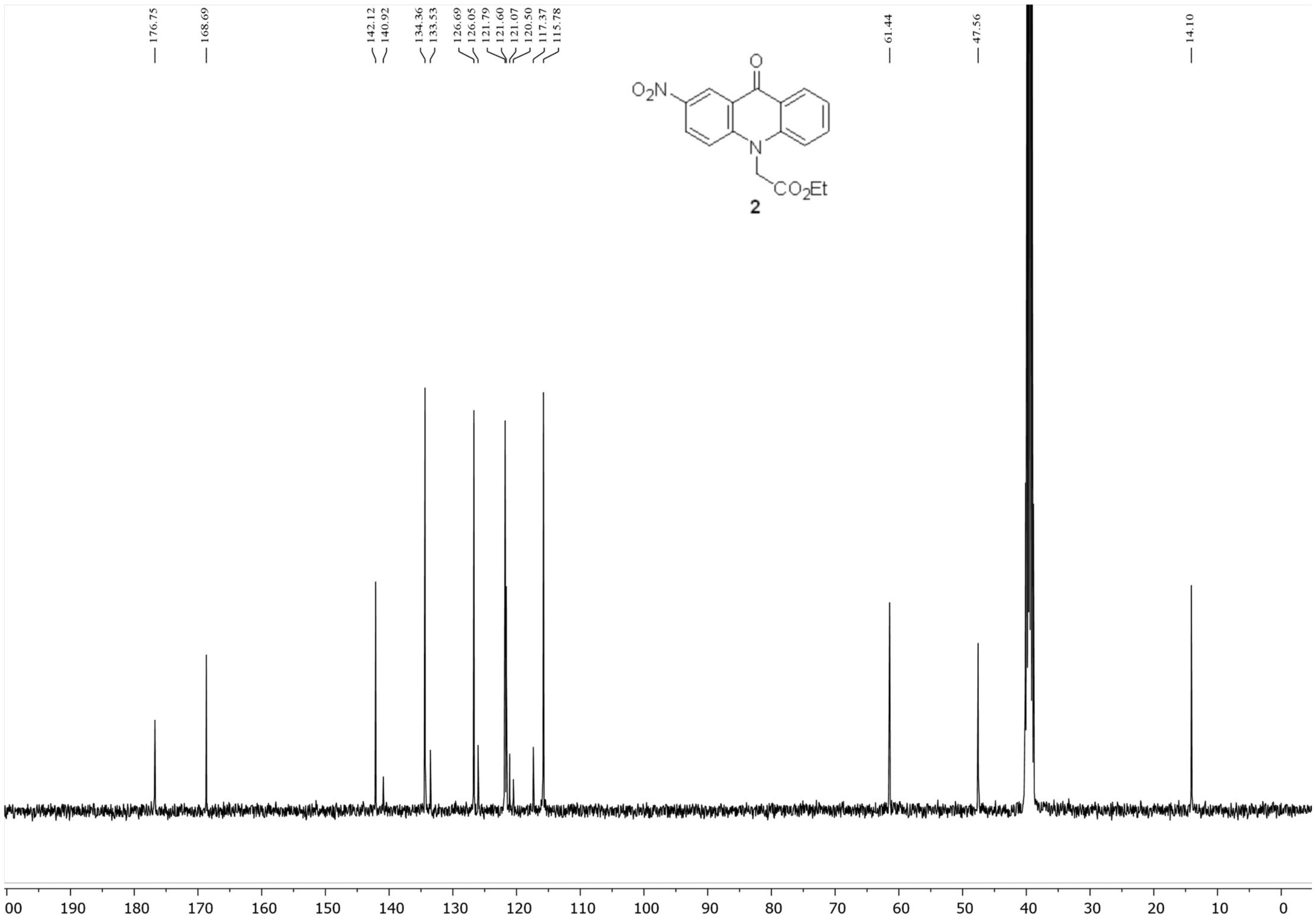

^{13}C spectrum of compound **2**

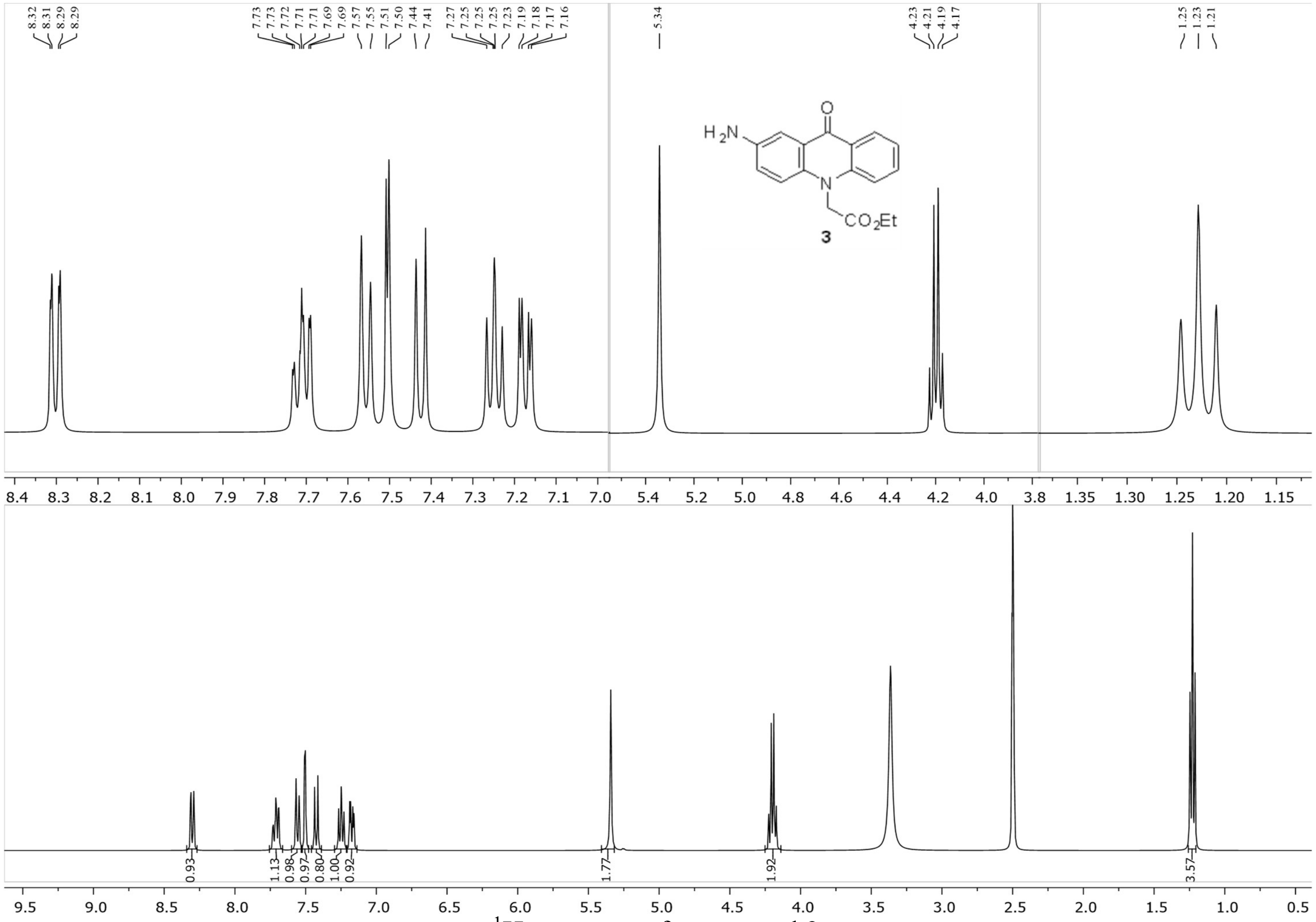

^{1}H spectrum of compound **3**

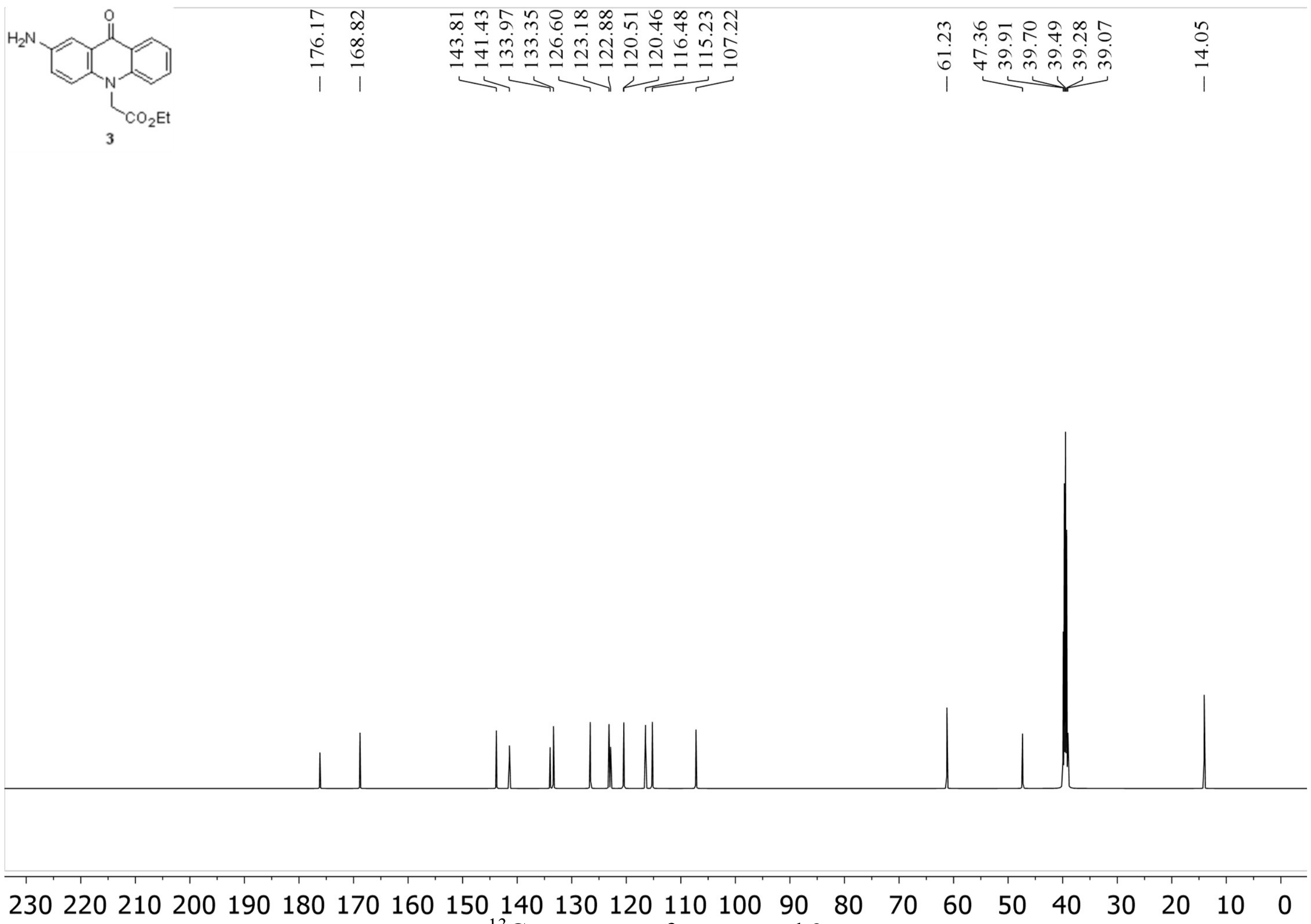

^{13}C spectrum of compound **3**

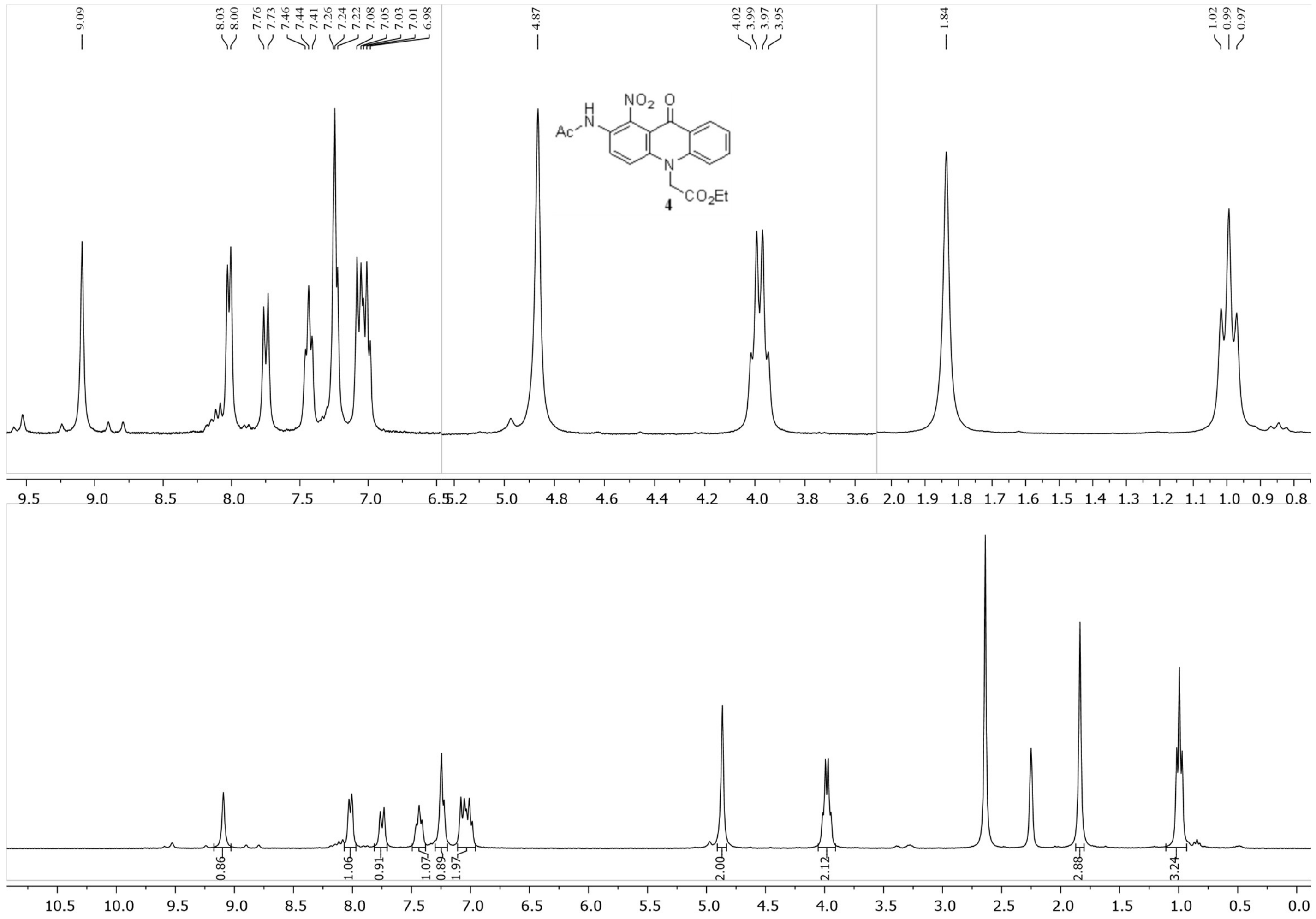

^{1}H spectrum of compound **4**

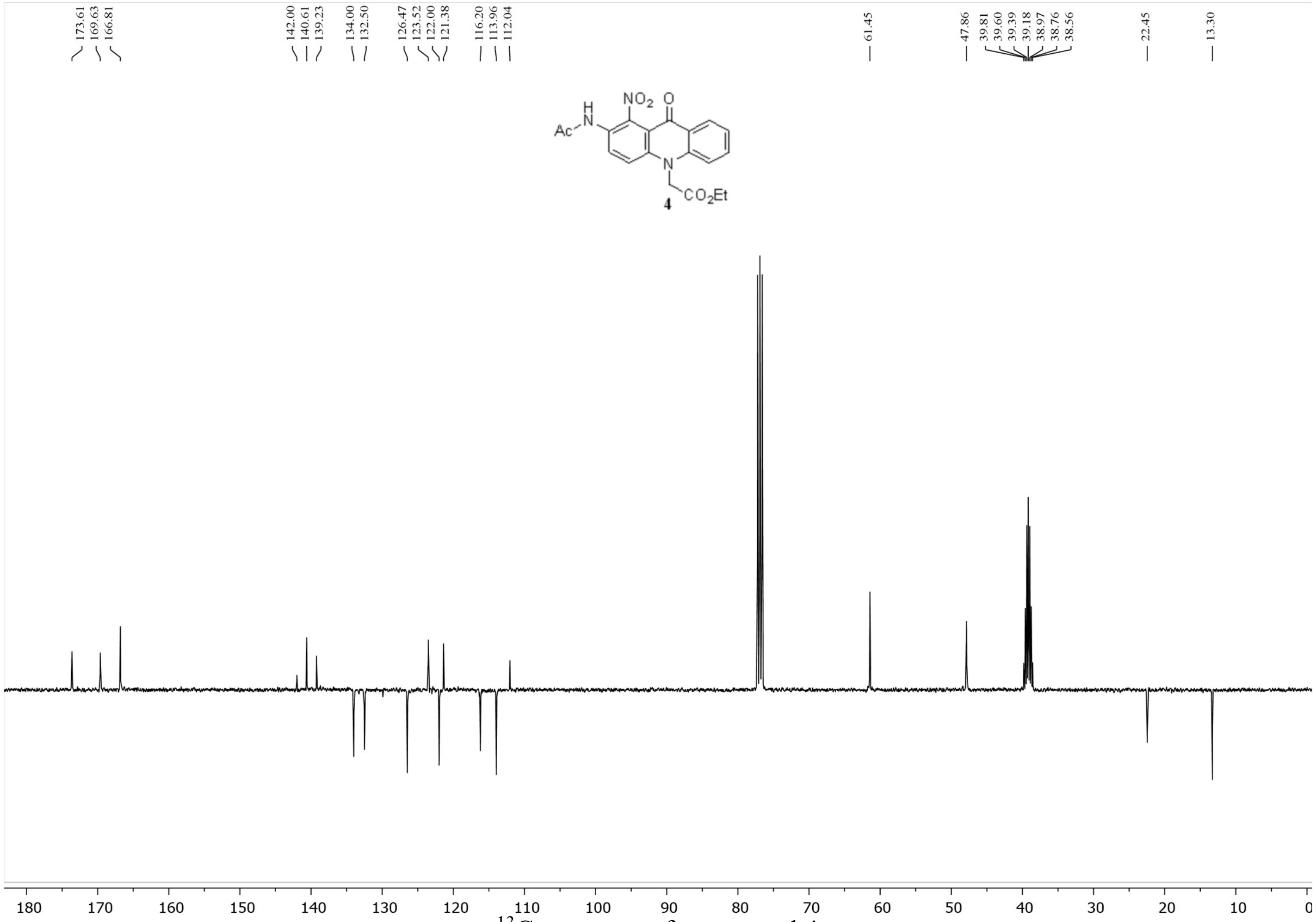

^{13}C spectrum of compound **4**

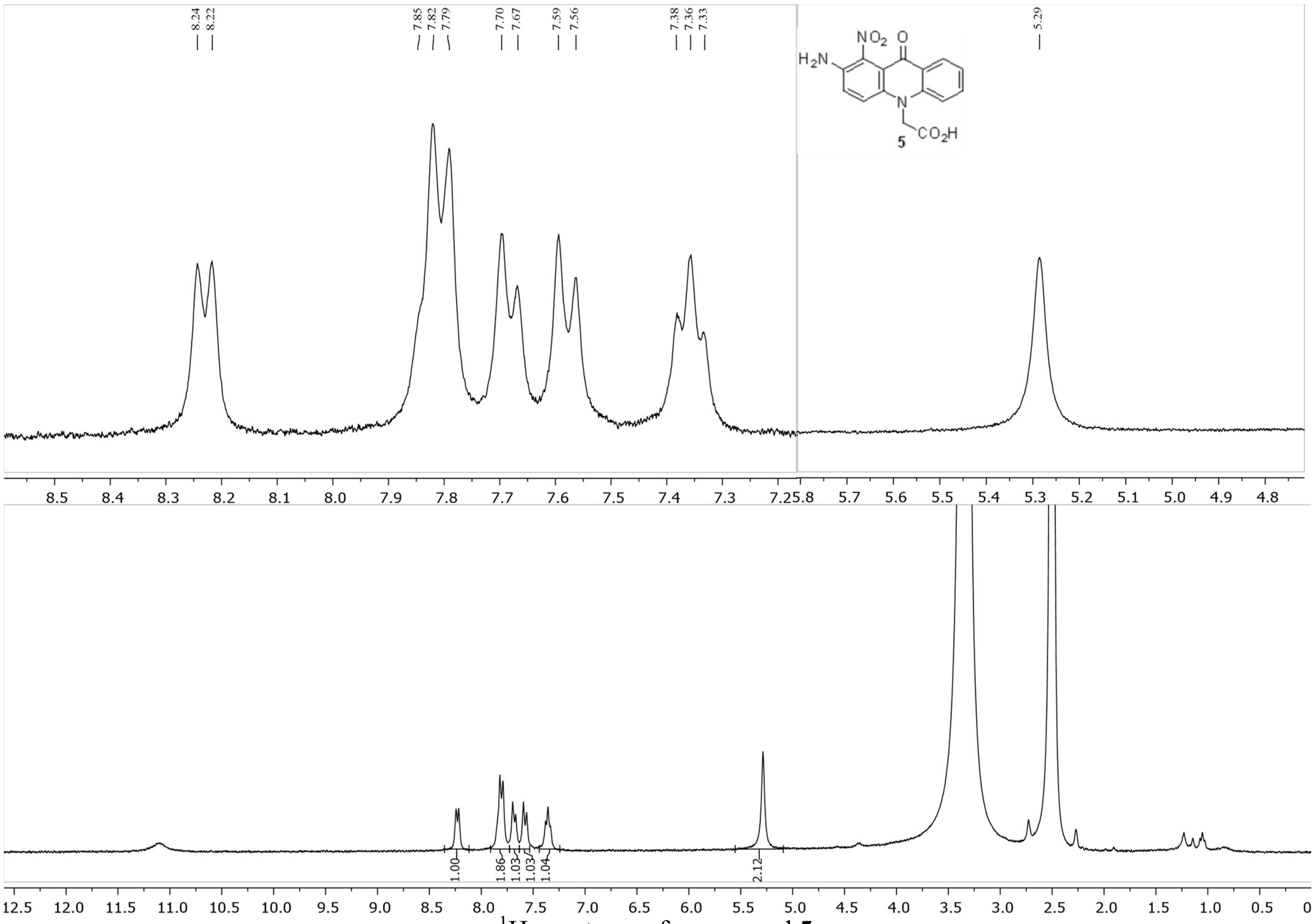

^{1}H spectrum of compound **5**

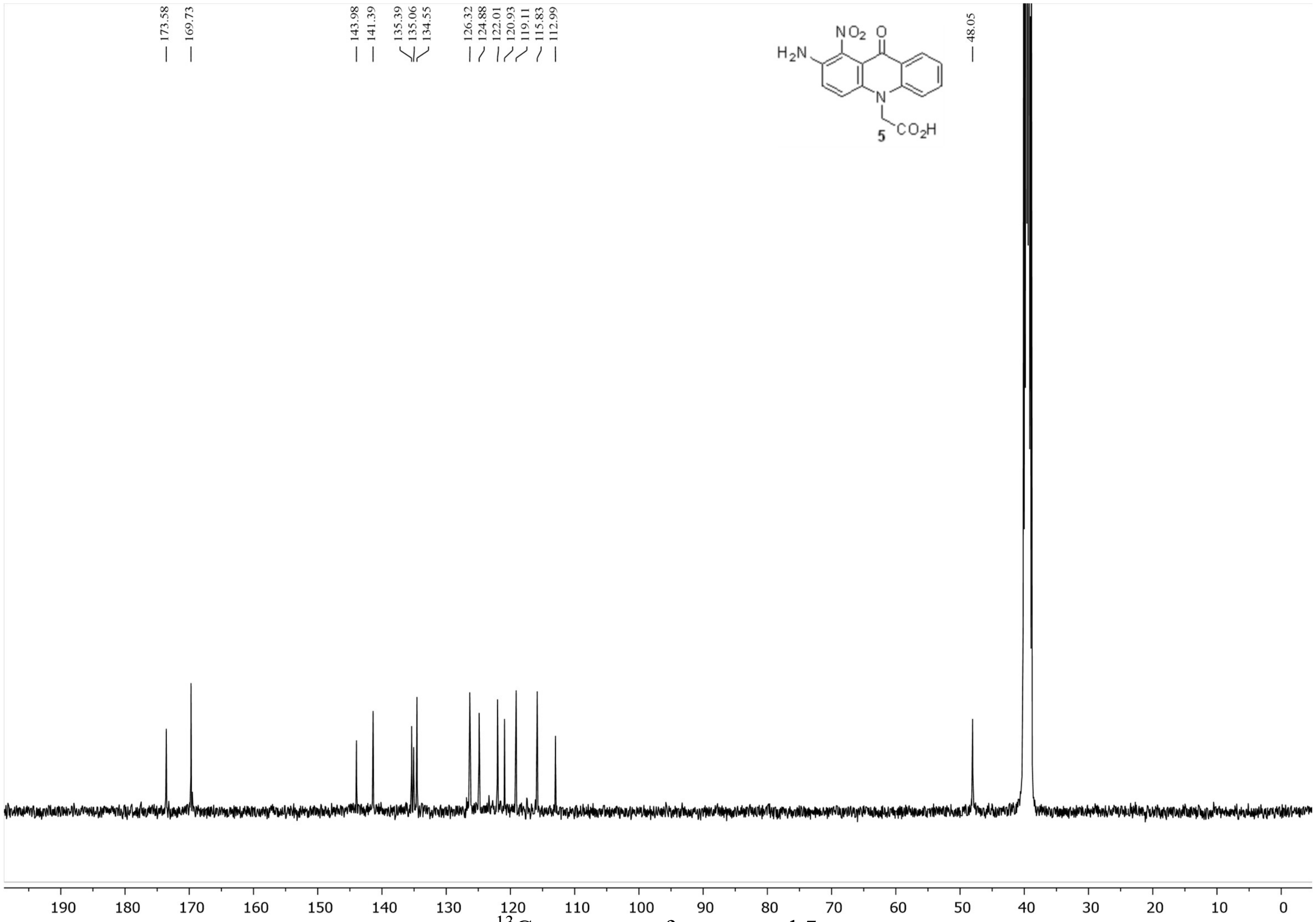

^{13}C spectrum of compound **5**

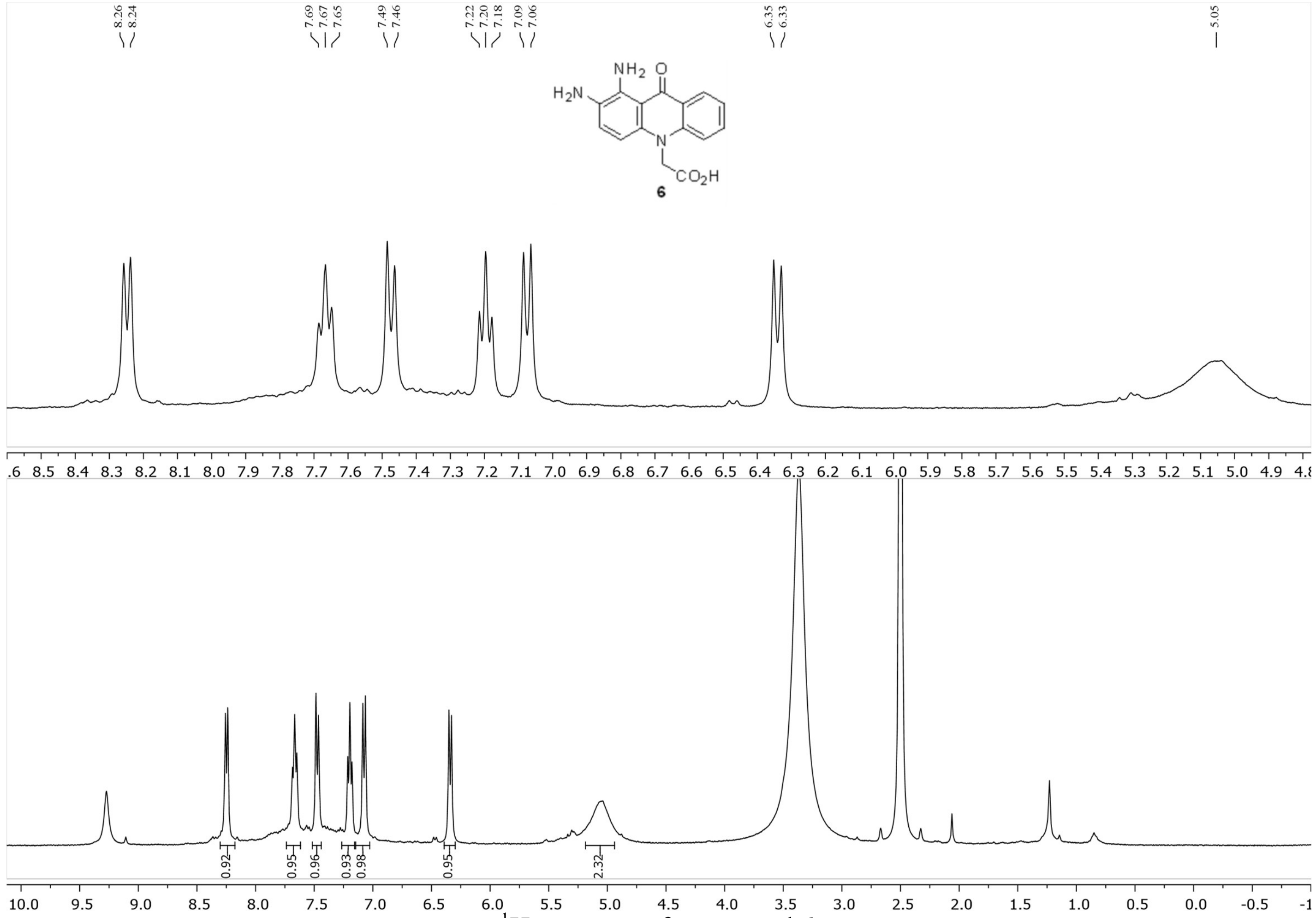

^{1}H spectrum of compound **6**

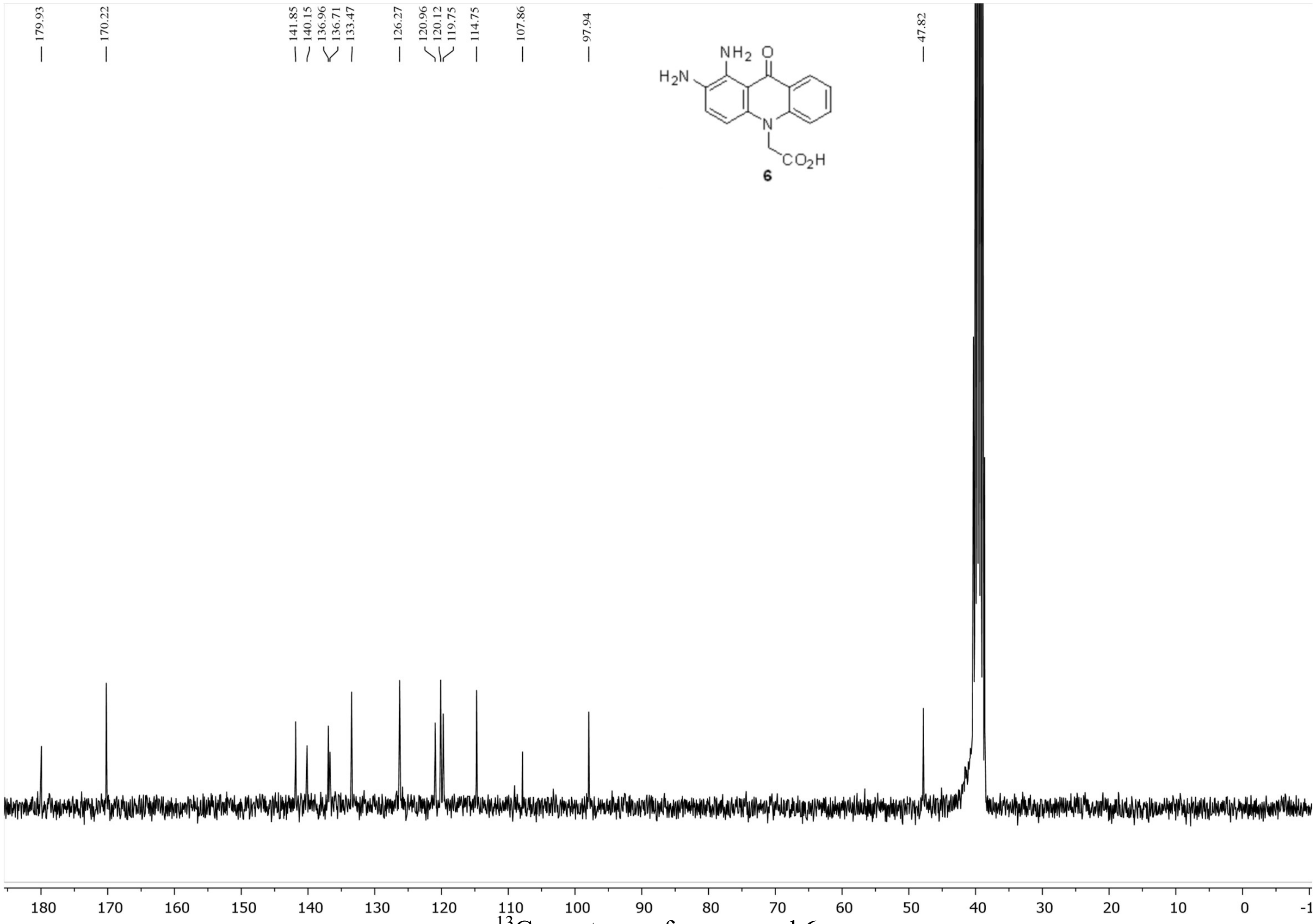

^{13}C spectrum of compound **6**

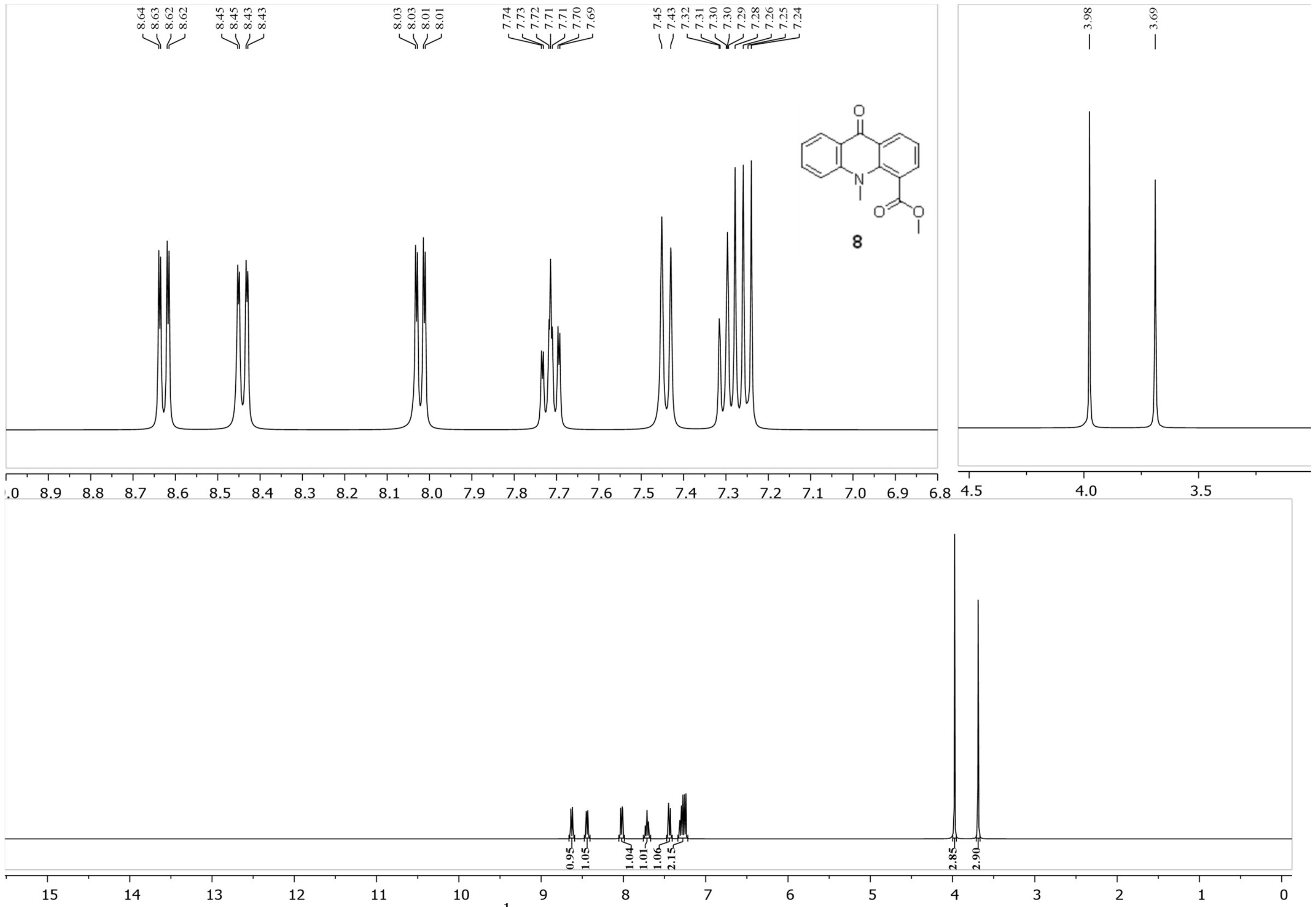

^{1}H spectrum of compound **8**

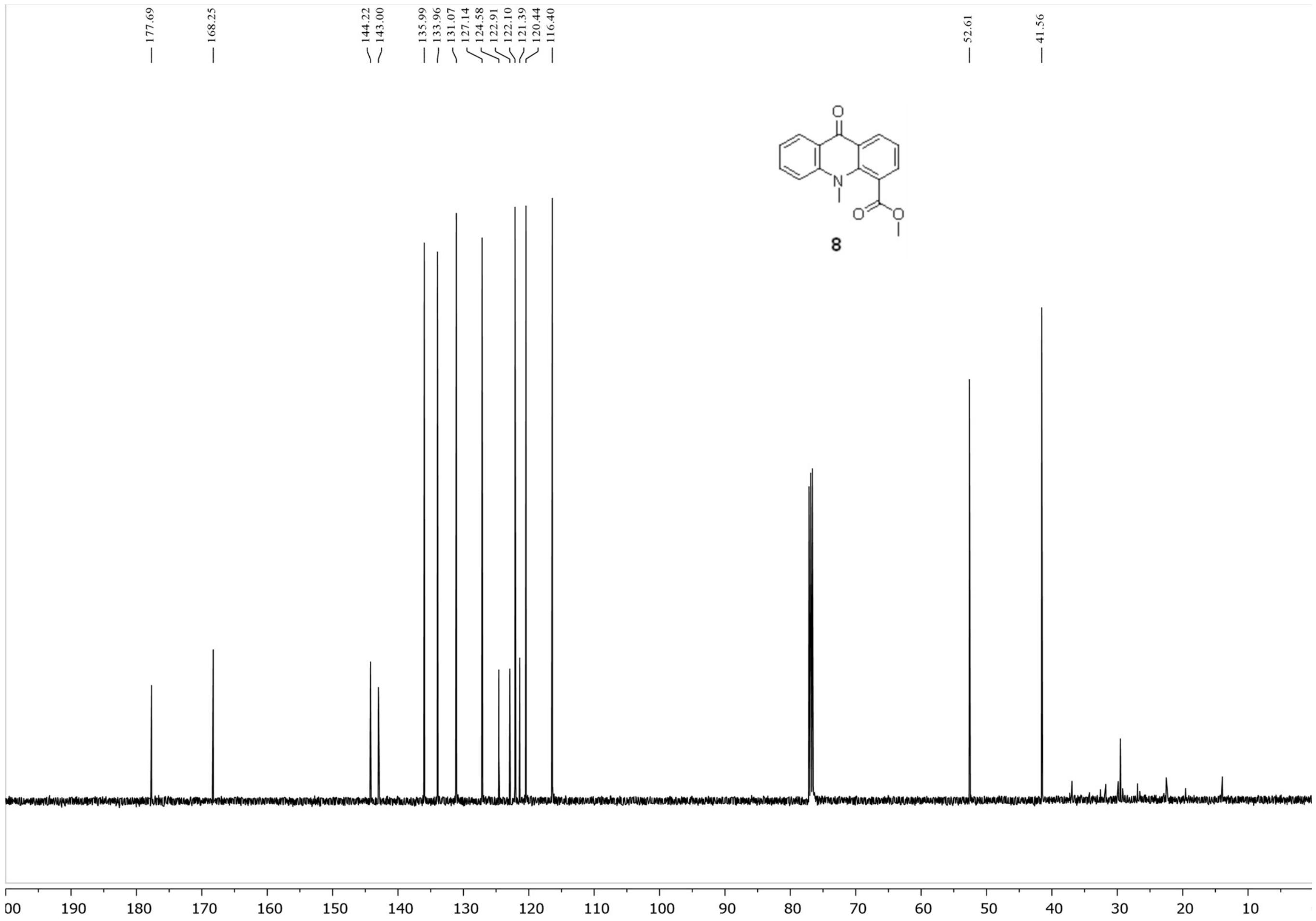

^{13}C spectrum of compound **8**

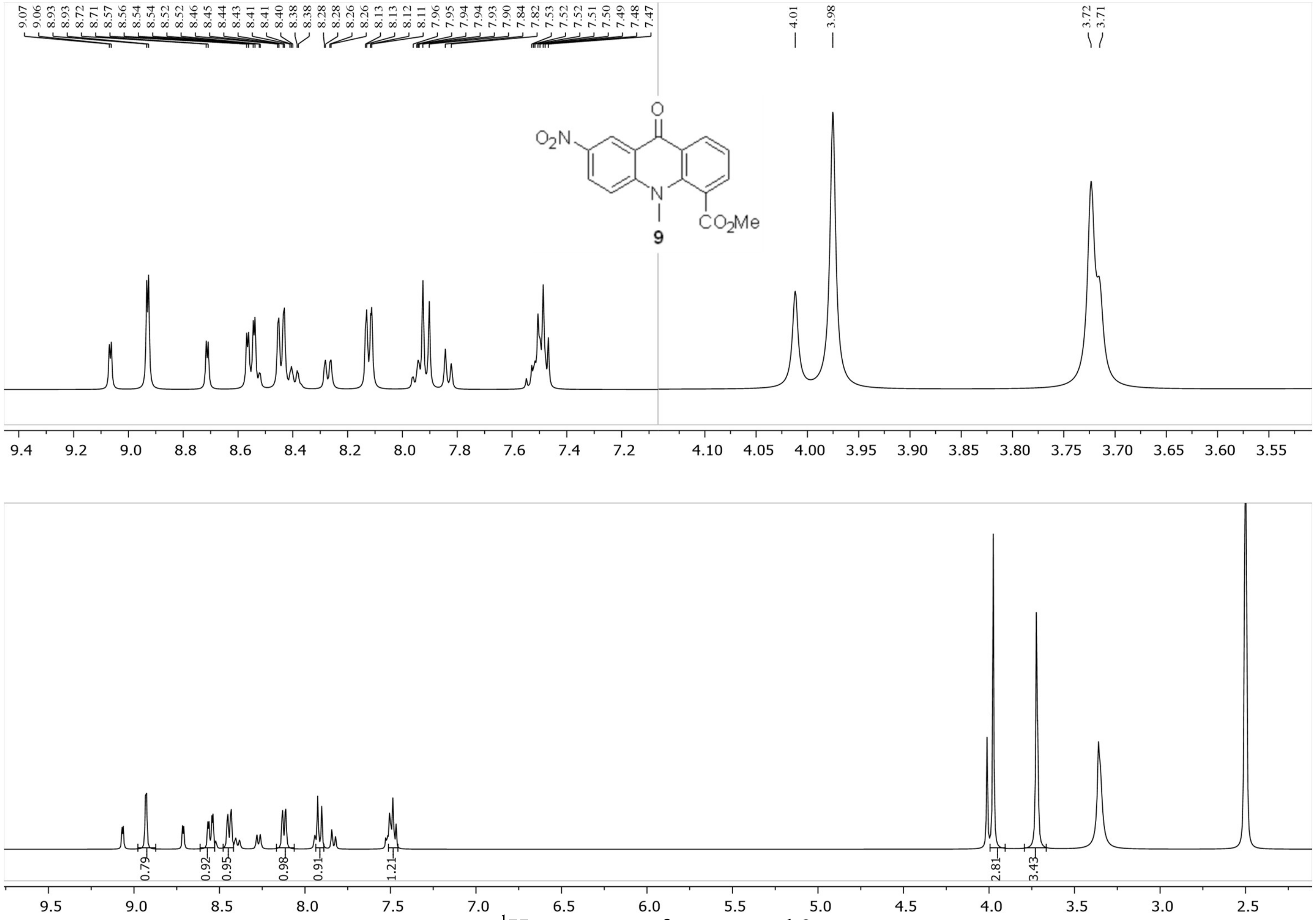

^{1}H spectrum of compound **9**

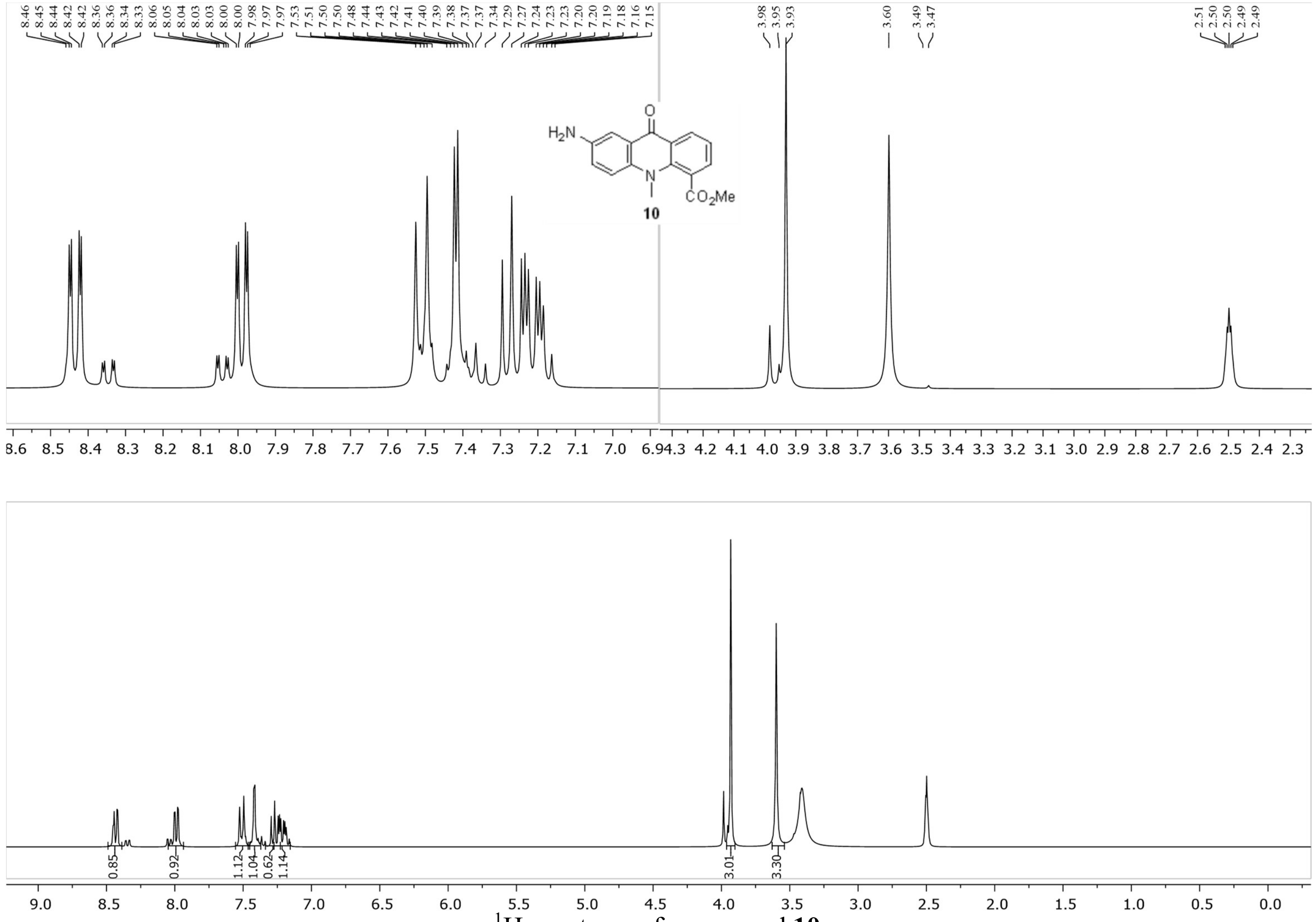

^{1}H spectrum of compound **10**

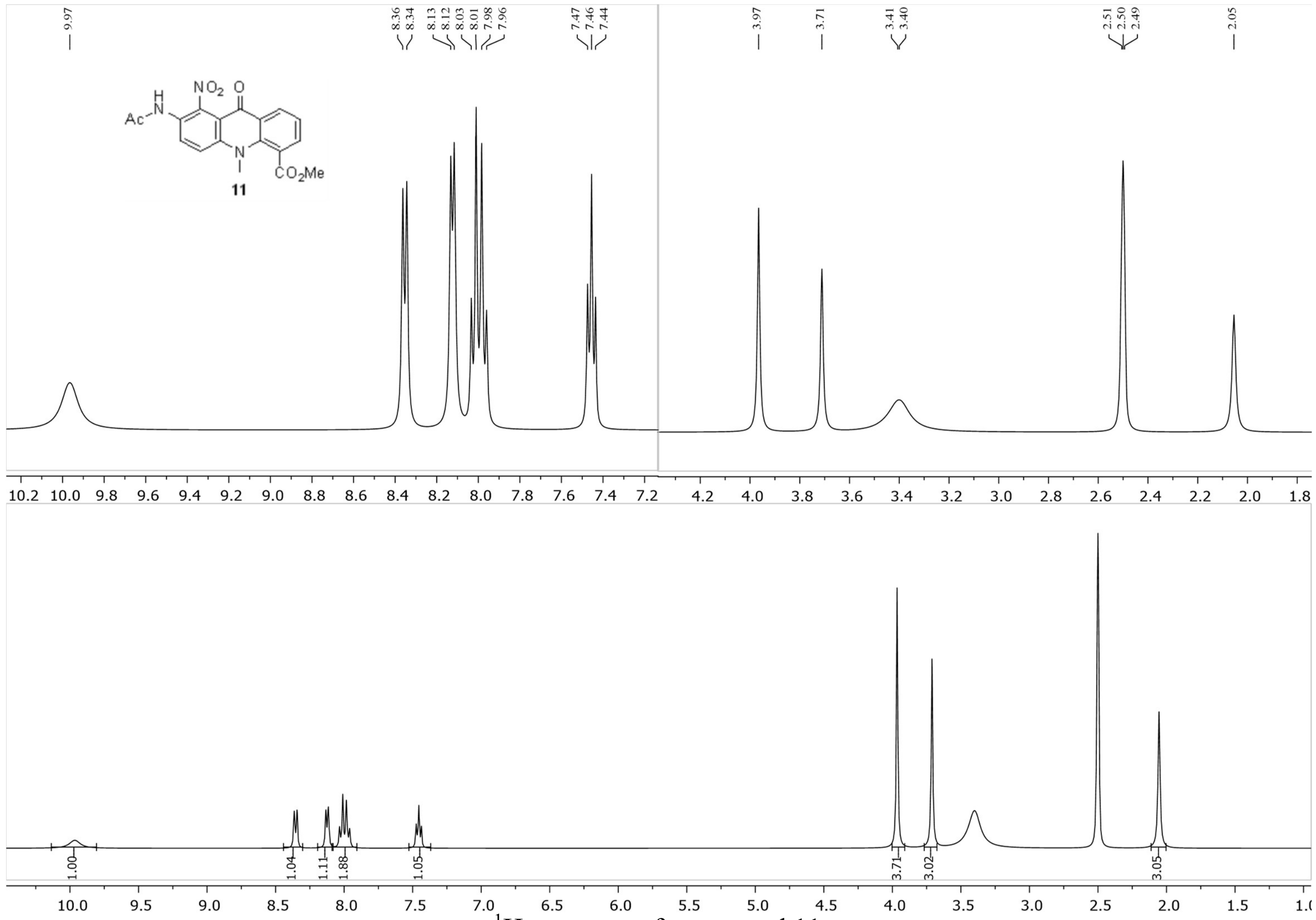

^{1}H spectrum of compound **11**

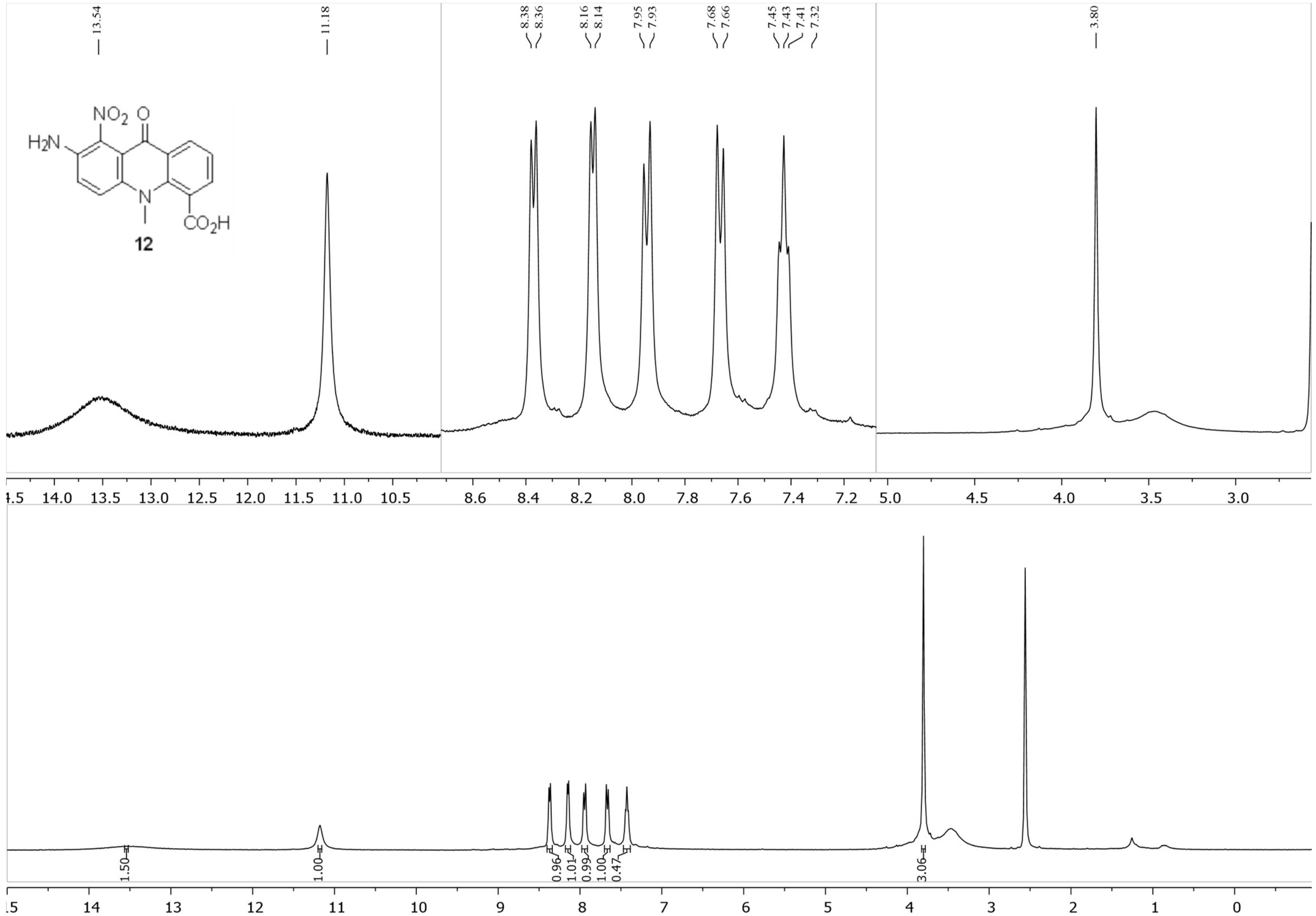

^{1}H spectrum of compound **12**

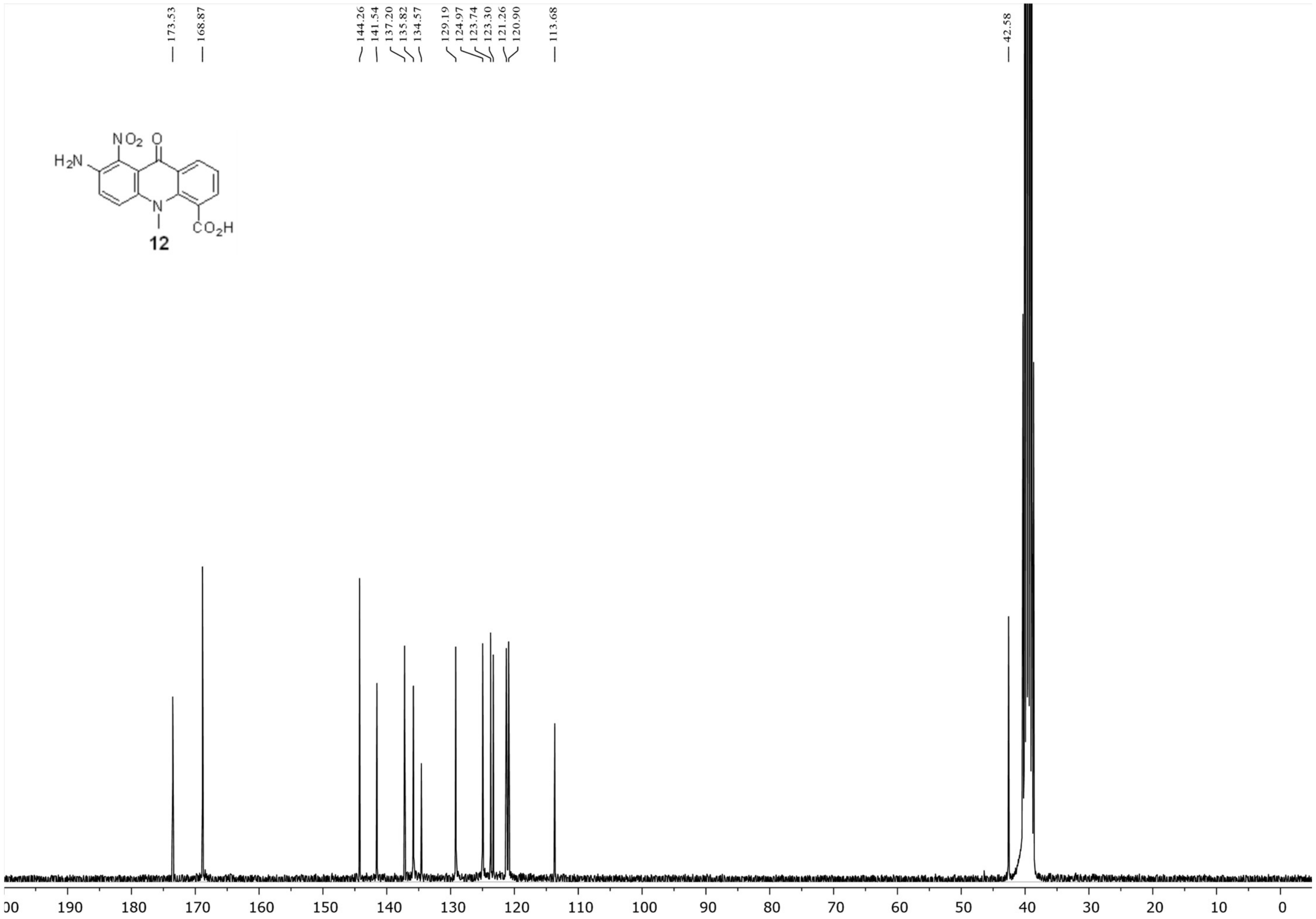

^{13}C spectrum of compound **12**

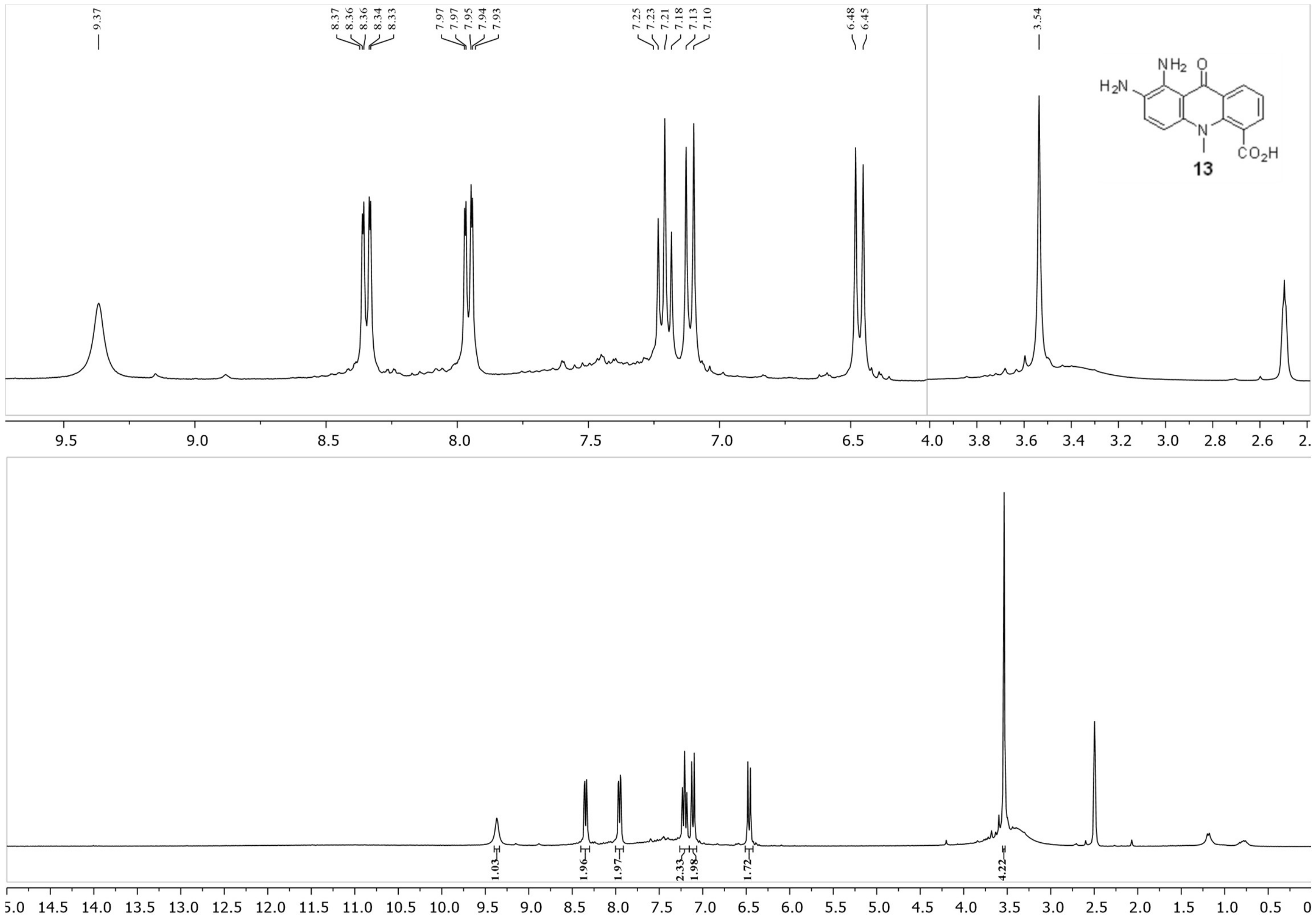

^{1}H spectrum of compound **13**

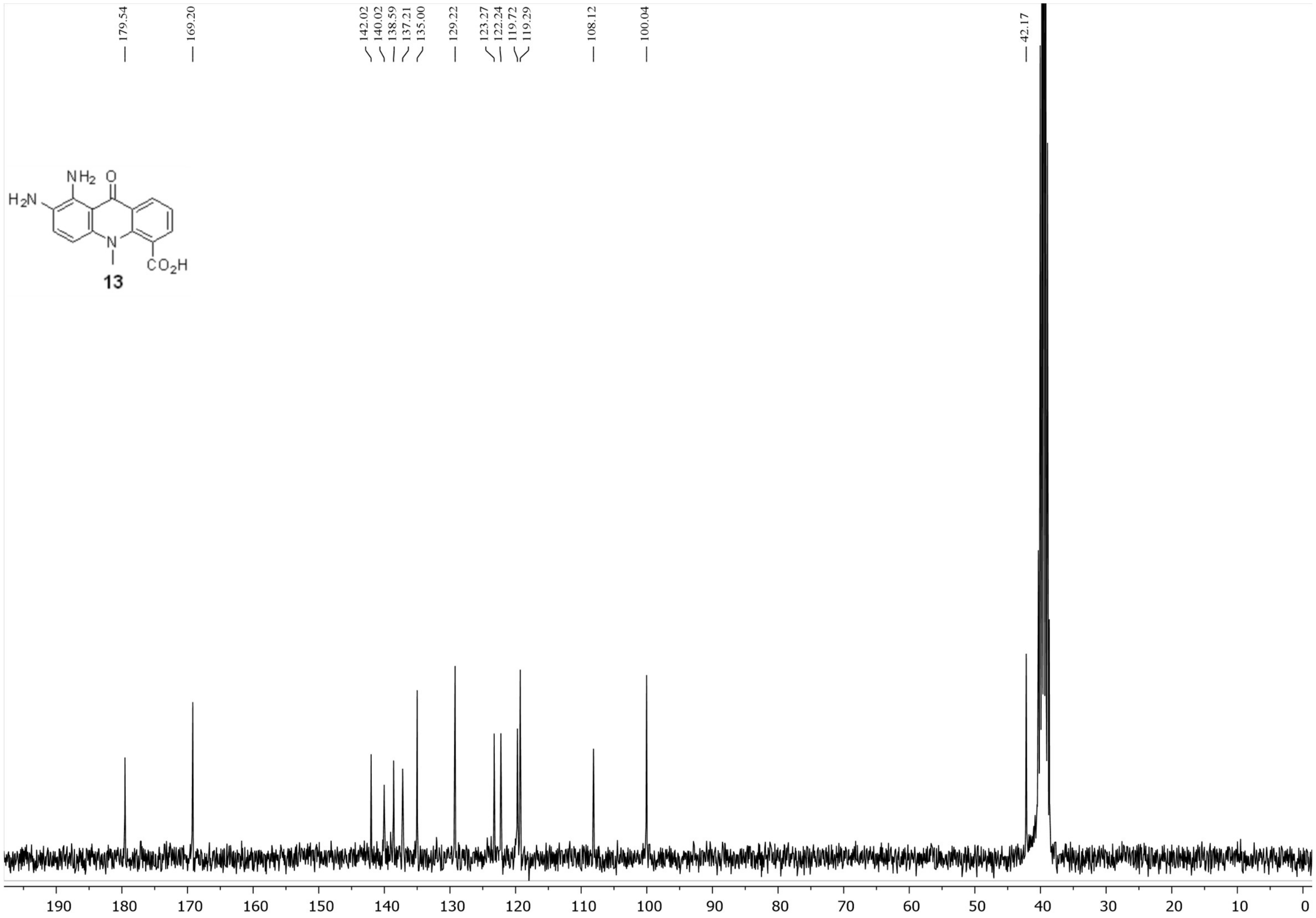

13

^{13}C spectrum of compound **13**

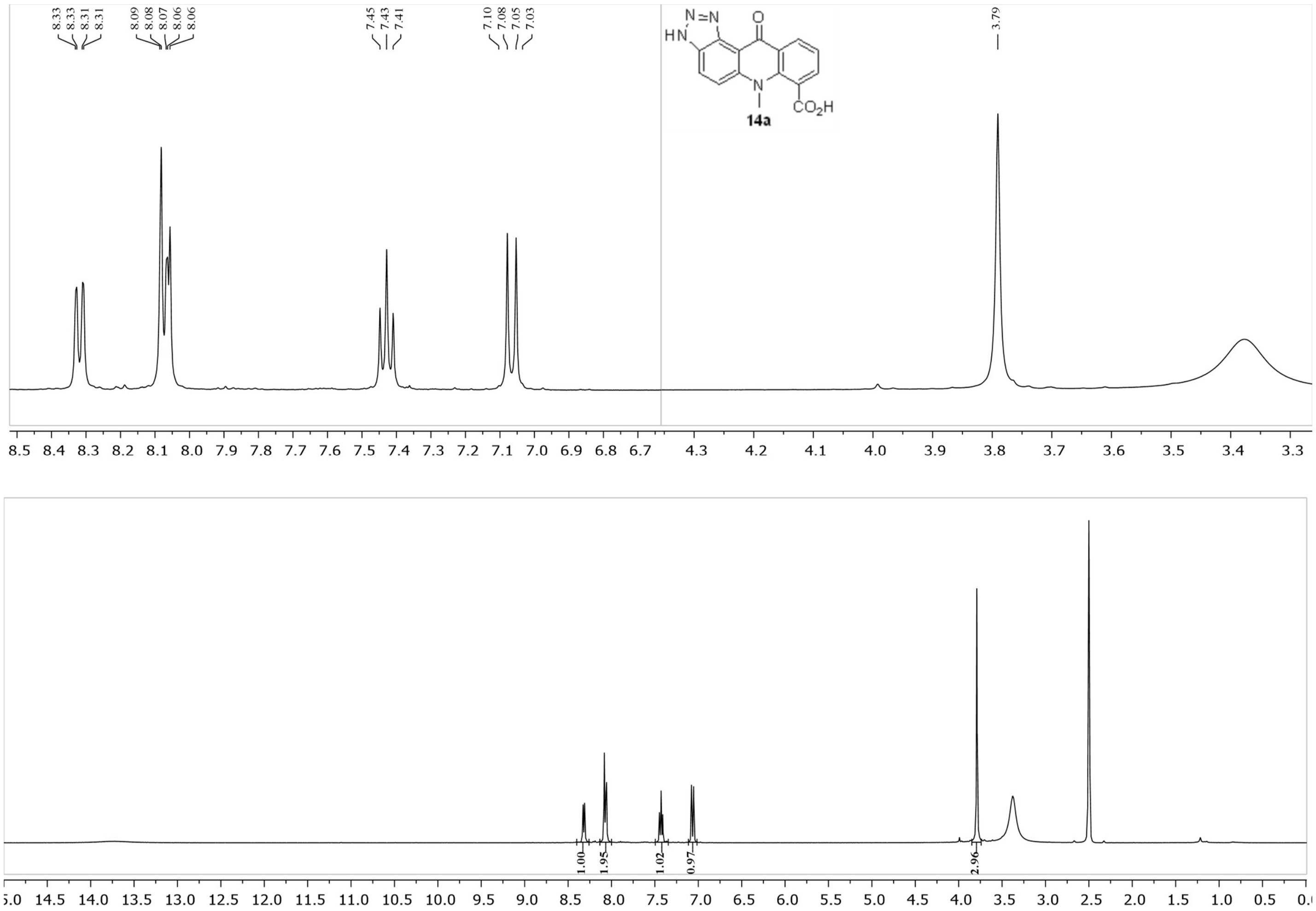

^1H spectrum of compound **14a**

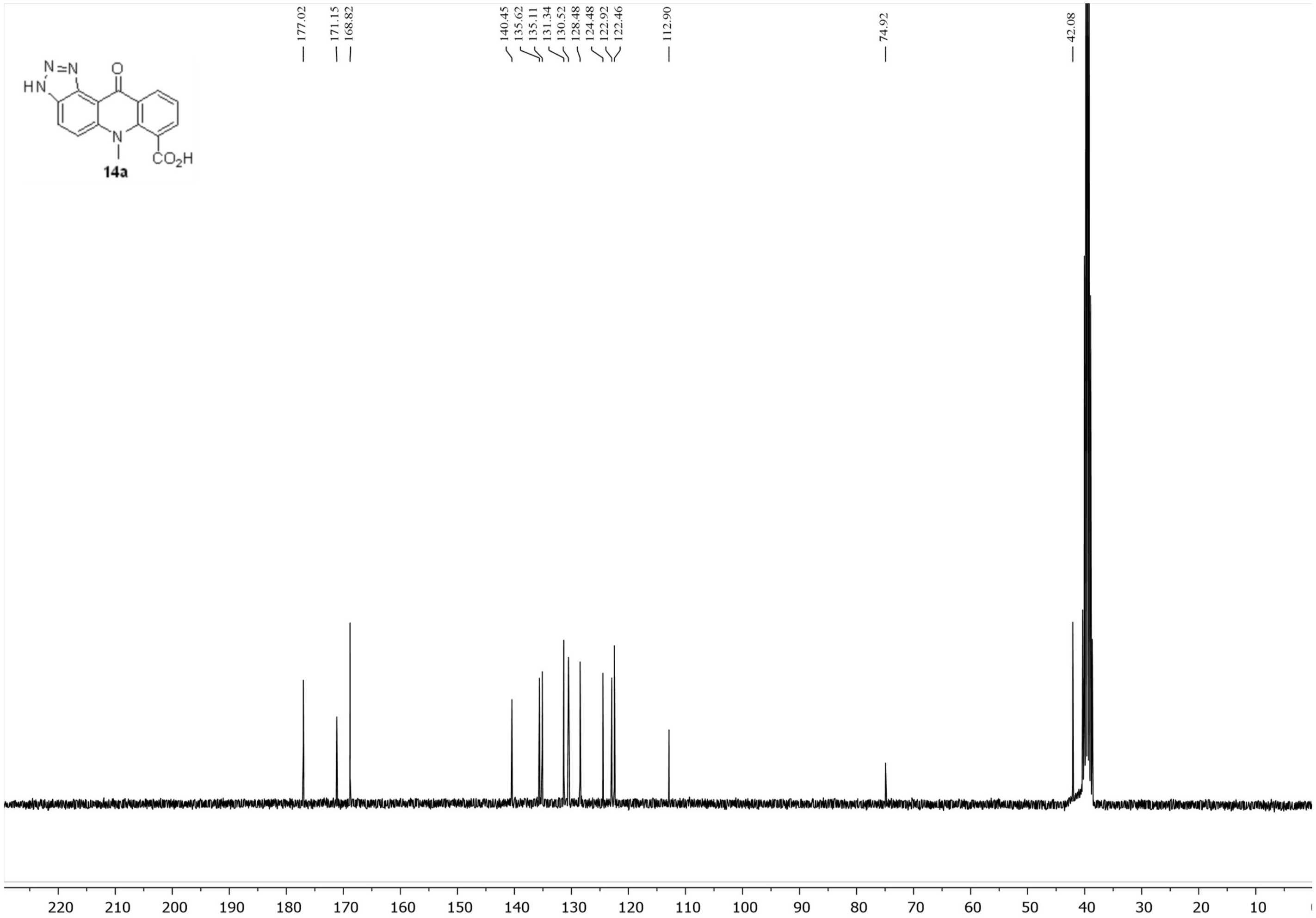

^{13}C spectrum of compound **14a**

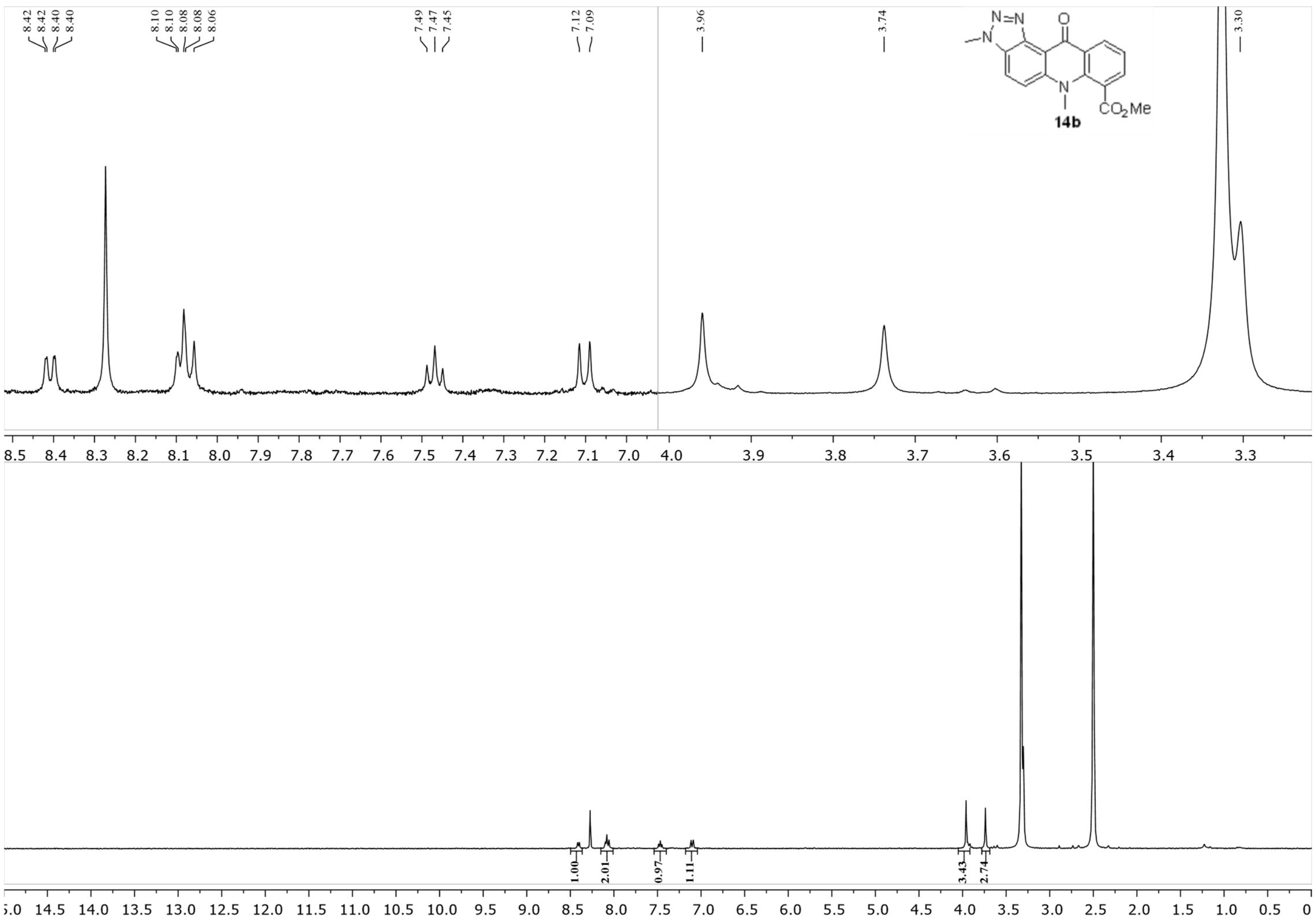

^{1}H spectrum of compound **14b**
